# Genetic mechanisms associated with floral initiation and the repressive effect of fruit on flowering in apple (*Malus* x *domestica* Borkh)

**DOI:** 10.1371/journal.pone.0245487

**Published:** 2021-02-19

**Authors:** Chris Gottschalk, Songwen Zhang, Phil Schwallier, Sean Rogers, Martin J. Bukovac, Steve van Nocker

**Affiliations:** 1 Department of Horticulture, Plant and Soil Science Building, Michigan State University, East Lansing, Michigan, United States of America; 2 Michigan State University Extension, East Lansing, Michigan, United States of America; Huazhong University of Science and Technology, CHINA

## Abstract

Many apple cultivars are subject to biennial fluctuations in flowering and fruiting. It is believed that this phenomenon is caused by a repressive effect of developing fruit on the initiation of flowers in the apex of proximal bourse shoots. However, the genetic pathways of floral initiation are incompletely described in apple, and the biological nature of floral repression by fruit is currently unknown. In this study, we characterized the transcriptional landscape of bourse shoot apices in the biennial cultivar, ’Honeycrisp’, during the period of floral initiation, in trees bearing a high fruit load and in trees without fruit. Trees with high fruit load produced almost exclusively vegetative growth in the subsequent year, whereas the trees without fruit produced flowers on the majority of the potential flowering nodes. Using RNA-based sequence data, we documented gene expression at high resolution, identifying >11,000 transcripts that had not been previously annotated, and characterized expression profiles associated with vegetative growth and flowering. We also conducted a census of genes related to known flowering genes, organized the phylogenetic and syntenic relationships of these genes, and compared expression among homeologs. Several genes closely related to *AP1*, *FT*, *FUL*, *LFY*, and *SPLs* were more strongly expressed in apices from non-bearing, floral-determined trees, consistent with their presumed floral-promotive roles. In contrast, a homolog of *TFL1* exhibited strong and persistent up-regulation only in apices from bearing, vegetative-determined trees, suggesting a role in floral repression. Additionally, we identified four *GIBBERELLIC ACID (GA) 2 OXIDASE* genes that were expressed to relatively high levels in apices from bearing trees. These results define the flowering-related transcriptional landscape in apple, and strongly support previous studies implicating both gibberellins and *TFL1* as key components in repression of flowering by fruit.

## Introduction

In many tree fruits and nuts, flowering follows a biennial cycle, with maximal and minimal flowering alternating yearly [1–3]. This phenomenon, termed biennial (alternate) bearing, is both an intriguing biological phenomenon and a significant limitation for the production of many horticultural crops. In commercial (domesticated) apple, similar to many other tree fruit species, flowering spans two growing seasons. In the first growing season, floral meristems initiate at the tips of condensed shoots called bourse shoots [4–7]. The floral meristems develop during the remainder of the growing season and arrest in a partially developed state before the winter dormant period. In early spring of the subsequent growing season, flowers complete development, culminating in bloom shortly after release from dormancy. This two-year cycle leads to an overlap between the period of fruit development (from the previous season’s flowers) and the period of floral initiation (current season). At least for domesticated apple, it is generally acknowledged that the presence of developing fruit inhibits floral initiation within the adjacent bourse shoot. Several ideas have been offered to explain how developing fruit might repress floral initiation. For example, gibberellins (GAs) have been shown to repress floral initiation in apple [8], and as developing fruit contain relatively high concentrations of gibberellins [9], it is thought that diffusion of GAs from the fruit to shoot apex could underlie floral repression [8]. It has also been hypothesized the biennial bearing results from diversion of photosynthate from the apex to the developing fruit due to the potentially higher sink strength of the fruit [3, 10].

The repressive effect of fruit should ultimately be reflected in expression of floral-promotive genes at the shoot apex. Previous studies have identified putative molecular components of the flowering pathway in apple based on apparent homology with well-studied flowering genes such as *APETALA 1* (*AP1*), *LEAFY* (*LFY*), *SUPPRESSOR OF CONSTANS* (*SOC1*), *FRUITFULL* (*FUL*), *FLOWERING LOCUS T* (*FT*), and *TERMINAL FLOWER 1* (*TFL1*). Homologs of the floral promoters *LFY* (designated *MdAFL2)*, *FUL (MdFUL/MdMADS2)*, and *FT (MdFT1)* were reported to exhibit increased expression within the apex during the anticipated period of floral induction [11–18]. In contrast, two homologs of the floral repressor *TFL1* (*MdTFL1-1/2*) were reported to exhibit rapidly decreasing expression either prior to or during the floral induction and initiation period [13, 14, 16–19]. During floral initiation, *MdFUL*, as well as homologs of the floral promoters *AP1* (*MdAP1a* and *MdAP1b*), and *SOC1* (*MdSOC1*), exhibited increasing expression [14, 16–18, 20]. Following floral initiation, the expression of homologs of *FT* (*MdFT2*), *LFY* (*MdAFL1*), and *AP1* (*MdAP1*) were either maintained at a relatively high level or were further increased coinciding with floral development [11, 13, 14, 16–18, 20]. During floral development and thereafter, *MdTFL1-2* expression increased [14, 18, 19]. Various homologs of *AP1*, *LFY*, *FT*, and *TFL1* have been additionally examined for flowering function by manipulating their expression in transgenic Arabidopsis or apple [11, 12, 16, 19, 21–24].

Relatively few studies have investigated the effect of fruit load on the expression of specific, presumed flowering genes in apple or other tree fruit species. In biennial-bearing avocado and citrus cultivars, *FT*-like genes were expressed in fully developed adult leaves during the period of floral initiation, and this expression was found to be significantly higher in the bearing year [25, 26]. However, in the biennial-bearing apple cultivar ‘Red Delicious’, *MdFT1* expression in the leaves was found to be similar between non-bearing and bearing years [18]. On the other hand, a PCR-based study suggested that the expression of a *MdFT2* was higher in apical buds of apple trees carrying a high fruit load compared with trees with no fruit [17, 27]. Haberman et al. [18] reported that *MdTFL1-1* expression in bourse shoot apices decreased during the course of the growing season, and that the decrease in *MdTFL1-1* expression was more rapid in trees carrying a high fruit load compared to low fruit load. In addition, they found that *MdTFL1-2* expression significantly increased relatively late in the season, but only in the high fruit load trees. This pattern was interpreted as suggesting that *MdTFL1-1* might play a role in maintaining the apex in a vegetative state early in the growing season, whereas *MdTFL1-2* might repress flowering in response to high fruit load [18].

Although these previous studies documenting gene expression in the apex have provided a solid blueprint for the advanced molecular study of flowering and alternate bearing in apple, they have focused on a limited number of anticipated landmark genes. In this study, as a subsequent step to understanding the genetic basis of floral repression by fruit in apple, we carried out an extensive census of flowering-related genes, a comprehensive analysis of gene expression in the bourse shoot apex during the transition to floral initiation, and evaluation of the effect of fruit load on the expression of flowering-related genes. Ultimately, this work should provide a deeper understanding of the endogenous mechanism(s) responsible for floral initiation and alternate bearing. This, in turn, may facilitate the development of approaches to control flowering in commercial operations, and the development of new cultivars less prone to alternate bearing.

## Materials and methods

### Plant materials, growth conditions and field experimental design

Field experiments were conducted at the Michigan State University (MSU) Clarksville Research Center (Field: 42°52’28.91"N, 85°16’8.15"W–Station: 42°52’24.20"N, 85°15’30.81"W) located in Clarksville, MI. Trees were managed in accordance with standard commercial practices for disease, insect, and weed control. ‘Honeycrisp’ trees had been established for five years as grafts on Nic 29^®^ rootstocks. The date of full bloom was defined as the date in which the maximum numbers of flowers were open but had not reached anthesis. Six trees were chosen that showed at least 80% bloom density, defined as the percentage of nodes on one-year-old shoots that showed flower clusters. For each tree, six branches, each between 4 and 6 cm diameter at the base, were selected and randomly assigned for apex collection dates (five branches) or for observation of flowering the following spring (one branch). Plants were randomly assigned as three replicate pairs, with each pair comprising one plant that was subjected to removal of all flowers, and one plant that was left untouched. Collections were made at 2 days after full bloom (DAFB), 15–17 DAFB, 35–38 DAFB, 49–52 DAFB, and 72–75 DAFB. On each collection date, dominant buds immediately subtending the position of flower clusters or former cluster position, or the apex of actively growing shoots originating from this position, were removed using a razor blade, immediately frozen in liquid N_2_, and transferred to storage at -80°C.

### Nucleic acid preparation, sequencing, and data analyses

RNA was isolated from frozen apex samples using the method of Gasic et al. [28] with the exception that spermine was substituted for spermidine in the extraction buffer, followed by a final ’clean-up’ step using a commercial kit (RNeasy Mini; QIAGEN, Germantown, MD). RNA quality and quantification was analyzed by the use of a Nanodrop 2000c (Thermo Scientific, Waltham, MA) and electrophoresis (2100 Bioanalyzer; Agilent, Santa Clara, CA). Library preparation and sequencing used the Illumina (San Diego, CA) platform and TruSeq platform with 101-b paired-end protocols, starting with 1 ug of total RNA from each sample. The raw sequence files were processed with fastq-mcf [29] using the parameters -t 0.10 -p 15 -l 20 -q 25 to remove adapter sequences, very short reads, and terminal bases with a Phred score below 25. The number of read pairs generated is shown in S1 Table.

#### Reference-based transcriptome assembly

Sequence reads were aligned to v1.1 of the GDDH13 reference sequence [30] and splice junctions were identified using the program HISAT2 (v.2.1.0) invoking the—dta-cufflinks and—un-conc-gz options [31]. The—un-conc-gz option was invoked to capture reads that failed to map to the reference genome. HISAT2 was operated using the default maximum and minimum mismatch penalties of six and two, respectively. Alignment metrics are shown in S1 Table. Transcript models were assembled using StringTie (v.1.3.3) using default parameters, including the -G option for use of a reference annotation as described [32, 33]. Transcript models generated for each sample library were reduced to a consensus set of transcript models using the StringTie–merge function. The program Cuffquant (v.2.2.1; included in the Cufflinks suite [34]) was then used to calculate sequence read counts for each transcript model, and significant differentially expressed genes and isoforms were identified by the use of Cuffdiff [35]. Metrics for assessing read mapping and transcriptome assembly were obtained using RNA-SeQC (v.1.1.8 [36]) and GFF utilities suite [37], respectively.

#### Identification of novel ‘Honeycrisp’ reference-based transcripts

Novel transcripts contained within the reference-based transcriptome were identified by comparing the reference genome gene models (retrieved from https://iris.angers.inra.fr/gddh13/the-apple-genome-downloads.html as gene_models_20170606.gff3) and the ‘Honeycrisp’ reference-based transcript models (S1 File) using the gffcompare (v.0.9.12) software package within the GFF utilities [37]. The resulting annotated gtf file was filtered for classification codes associated with non-isoform-like transcript features and/or assembly errors (classification codes: e, i, o, u, x, y) and removal of transcripts with lengths <200b. This subset of transcripts was then analyzed for protein-coding capacity using the software programs CPC2 (beta version [38]), PLEK (v.1.2 [39]), and CPAT (v.1.2.4 [40]). For CPC2 and CPAT, the coding potential probability was set to ≥0.5 to assign a transcript as coding and ≤0.5 as noncoding/ambiguous. For PLEK, the coding or noncoding/ambiguous determination was assigned by the program’s default parameters. The final coding definition of a transcript was based on an agreement between at least two of the programs. Detailed transcript information can be found in S2–S4 Tables.

#### *De novo* assembly of unmapped reads

Reads that were unmapped by HISAT2 were assembled into contiguous sequences using the Trinity *de novo* assembler (v.2.4.0) with default settings [41]. The resulting FASTA file (S2 File) containing the *de novo* assembled transcripts was then used as an input to construct consensus gene models using the python program Trinity_gene_splice_modeler.py provided by the Trinity suite (S3 File). The python script produced a consensus FASTA file containing gene models and a corresponding GTF file. The unmapped read files were then realigned to the consensus gene FASTA file using HISAT2 invoking the—dta-cufflinks options and using the previously generated GTF file. Alignments were then processed through the same Cufflinks pipeline used in the referenced-based transcriptome assembly. The initial output comprised ~250,000 sequences corresponding to ~92,000 distinct loci. Because most output sequences appeared to be sequencing or assembly artifacts, we limited further consideration to contigs representing putative transcripts that were likely to be strongly expressed (upper 10th percentile based on FPKM, and expressed in at least three samples) and that had coding potential (determined as described above for novel reference-based transcript models) (S5–S7 Tables).

#### General transcriptome annotation

Transcript sequences were annotated based on sequence homology to Arabidopsis open reading frame translations (TAIR10; TAIR10_pep_20101214_updated 2012-04-16, [42]) using the BLASTx module from NCBI [43] with an Expect (E)-value cutoff of 1e^-11^. Homologous sequences were then used as queries to identify similar transcripts within the ‘Honeycrisp’ transcriptome, using the tBLASTx module. Gene model sequences generated from the *de novo* assembly were annotated by aligning sequences to the nr NCBI database (downloaded on 2018-09-18, [44]) and the ‘Honeycrisp’ reference-based transcriptome using the BLASTx and BLASTn modules, respectively. A minimum E-value of 1e^-10^ and a max_target_seqs of 1 were used.

#### Data and protocol accessibility

Raw sequence libraries can be downloaded from the NCBI Short Read Archive under biosample SAMN04239699. Our constructed reference-based transcriptome annotation (S1 File), *de novo* transcript FASTA and annotation (S2 and S3 Files), phylogenies of all of the flowering genes, and differential expression data files (S4 and S5 Files) can be retrieved from the Dryad repository https://doi.org/10.5061/dryad.fn2z34tr5. Computation protocols used in this study can be retrieved from the Protocols.io repository dx.doi.org/10.17504/protocols.io.bp54mq8w.

#### Identification of apple flowering genes

To identify potential homologs of flowering genes, we indexed genes from Arabidopsis (TAIR10) annotated with potential roles in flowering: Gene Ontology terms 0048438 (’floral whorl development’), 0009908 (’flower development’), 0009910 (’negative regulation of flower development’), 0009911 (’positive regulation of flower development’), 0048578 (’positive regulation of long-day photoperiodism, flowering’), 0010220 (’positive regulation of vernalization response’), 0009909 (’regulation of flower development’), 0048510 (’regulation of timing of transition from vegetative to reproductive phase’), 0010321 (’regulation of vegetative phase change’), 0010228 (’vegetative to reproductive phase transition of meristem’), 0010048 (’vernalization response’), and 0010093 (‘specification of floral organ identity’). This set of 437 genes was manually curated to omit those without strong functional evidence for a direct role in flowering. The curated subset contained 180 genes. Conceptual translations of the corresponding representative gene models were obtained from TAIR (TAIR10_pep_20110103_representative_gene_model_updated) and used as queries to search open reading frame translations of our mapped-assembled and *de novo*-assembled transcript models (BLASTp) using an E-value cutoff of 1e^-12^. The open reading frame translations of the Honeycrisp assembled transcript models were identified using TransDecoder (v.5.5.0; https://github.com/TransDecoder). All identified transcript translations were then used as queries to search the Arabidopsis representative gene model translations. Those transcripts that reciprocally identified their original Arabidopsis query were defined as reciprocal homologs. For phylogenetic analyses of the 16 intensively studied flowering gene families, we considered only the 25 highest-scoring apple transcript translations and only the 25 highest-scoring Arabidopsis gene translations identified with each apple sequence query. Phylogenetic trees were then constructed using the ETE3 toolkit (v.3.1.1) build function invoking the standard_fasttree workflow under default settings [45–47]. Collinearity among identified flowering genes was performed using the MCScanX toolkit following the manual’s instructions and the use of default parameters [48]. Graphics to illustrate the collinear relationships between homeologous chromosomes and flowering genes identified by MCScanX were generated using Circos (v.0.69–6) program package [49].

#### Gene expression analysis

Estimated expression levels for homologs of flowering-related genes/transcripts were obtained from Cuffnorm and Cuffdiff output. Heat maps were created and expression profiles were clustered using R statistical software (v.3.5.2 [50]) and the cummeRbund (v.2.24.0 [51]) package. Expression profiles of homologous flowering-related genes that exhibited significant changes in expression were clustered using a *K*-means approach by the csCluster command of cummeRbund. Cluster expression pattern was then defined by the general trend of the modal expression pattern. Venn diagram of differentially expressed genes was created using an online tool (http://bioinformatics.psb.ugent.be/webtools/Venn/). Co-expressed gene modules were identified using the weighted gene co-expression network analysis (WGCNA) R package [52], following the analysis methodology outlined by Zhang and Horvath [53] and using normalized gene expression (FPKM) as calculated by cufflinks.

#### TaqMan® qRT-PCR

Confirmation of *MdTFL1* gene expression was determined using a two-step quantitative polymerase chain reaction (qRT-PCR). Primers and probes were designed from sequences assembled in our transcriptome and aligned to apple nucleotide sequences maintained by the NCBI (taxid: 3750). The primers and probes were designed in a previous study [54] and were based on the specificity to selected target sequence and overlapped of an exon junction (S8 Table). An apple homolog of *ACTIN* served as an internal control. The reactions were performed using an Agilent Technologies Stratagene Mx3005P (Santa Clara, CA) qPCR machine with cDNA derived from the RNA samples prepared for RNA-seq. Each reaction consisted TaqMan^TM^ Gene Expression Master Mix (10 μl), 5x diluted cDNA template (2 μl), forward (1 μl) and reverse primers (1 μl), and probe (1 μl) for *ACTIN*, the primer-probe assay for the gene of interest (1 μl), and ddH_2_O (4 μl). The thermal profile for TaqMan^TM^ assay followed the instructions provided with the Agilent machine.

## Results and discussion

### Effects of reducing fruit load on floral initiation

**Fig 1:**
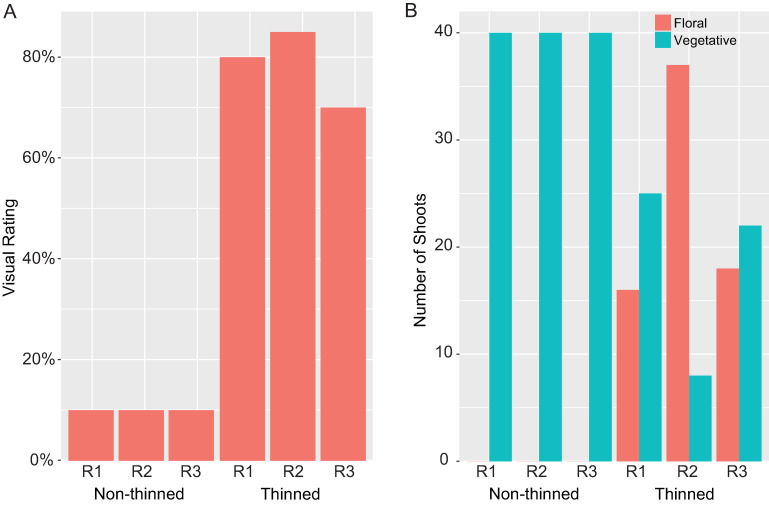
Effect of flower removal on subsequent-year flowering. At full bloom, trees were thinned of flowers or were left non-thinned, and the fraction of flower-bearing shoots, relative to total shoots arising from spur structures, was evaluated the following spring. (A) Visual estimation of floral density. The rating scale extends from 0% (no obvious flowers) to 100% (abundant flowers). Visual density was estimated by two, independent, trained observers (correlation p value < 0.05). (B) Quantification based on sampling a minimum of 40 spur shoots designated for evaluation prior to flower removal. Graphs show the results from three biological replicates (R1-R3).

As a physiological and molecular model for biennial bearing in apple, we focused on the popular commercial cultivar ‘Honeycrisp’, which can exhibit extreme biennial tendency under production conditions [55]. At full bloom in early spring, we selected three paired sets of trees with high bloom density and removed all flowers from one tree from each pair. This floral thinning treatment had a strong effect on initiation of new flowers, as evidenced by observed bloom density in the spring of the second year of the study (Fig 1A). Those trees that were thinned of flowers produced floral shoots at an average of ~52% (range 39–82%) of potential flowering nodes, whereas the non-thinned control trees produced almost exclusively vegetative shoots (Fig 1B).

### Transcriptome assembly and characterization

We sampled the bourse shoot apex from the thinned and non-thinned trees at approximately 2, 15, 35, 50, and 70 DAFB. Dissected apices were subjected to high-throughput RNA-based sequencing, yielding a total of ~390 million paired reads. These were aligned to a recently published reference genome sequence (GDDH13 v.1.1) assembled from a doubled-haploid individual generated from ‘Golden Delicious’ [30]. We obtained a mean alignment rate of 90.1%, with 96.3% of the aligned reads mapping within annotated intragenic regions (S1 Table). Transcript models were then assembled from aligned reads using the StringTie transcript assembler [32, 33].

**Fig 2:**
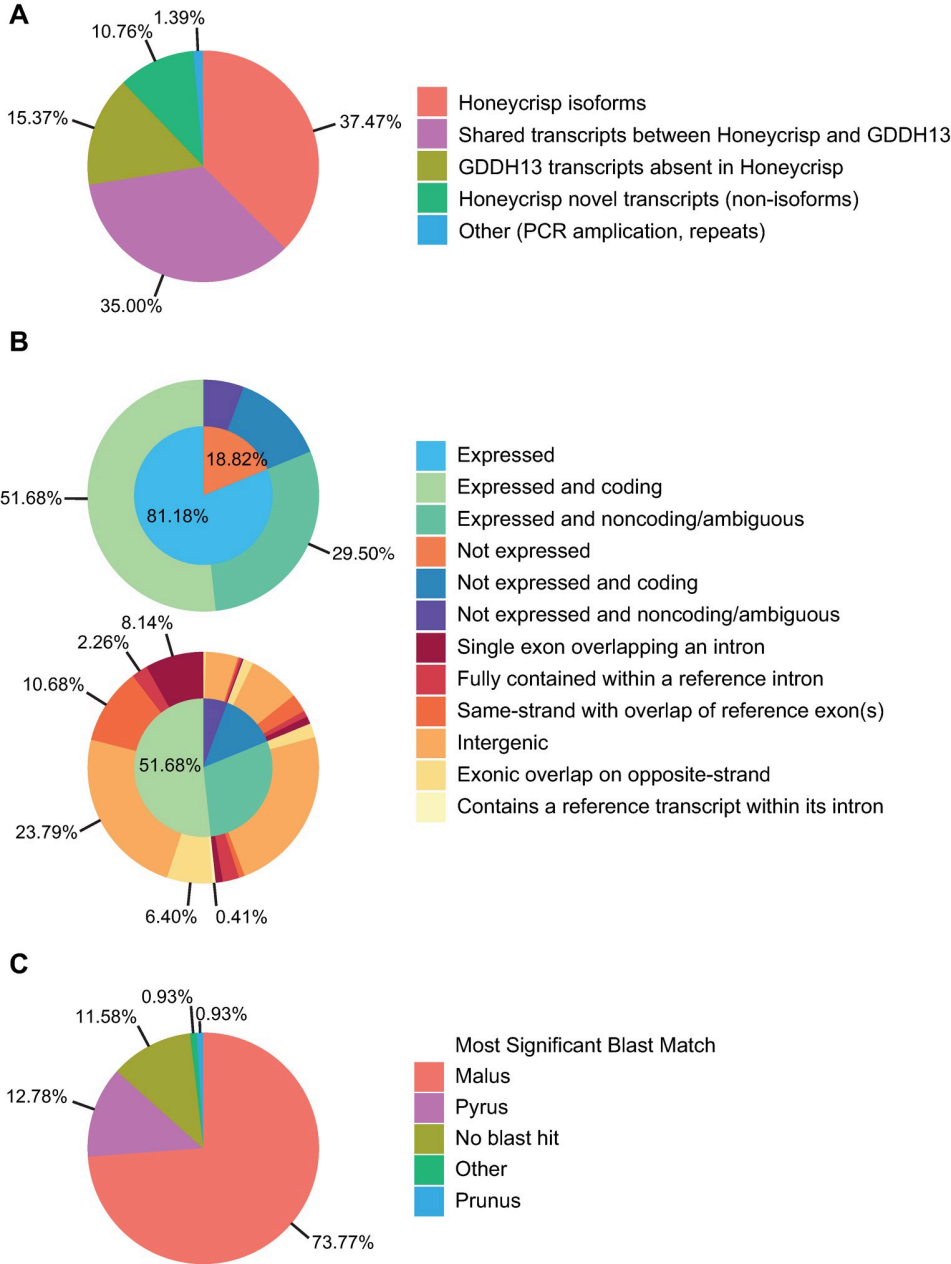
Characterization of the reference-mapped, assembled ’Honeycrisp’ transcriptome. (A) Assignment of transcripts. The gffcompare program was used to compare and classify the genomic organization of transcripts between the GDDH13 reference and ‘Honeycrisp’ transcriptomes (B) Characterization of ‘Honeycrisp’ novel transcripts by expression state, coding potential, and genomic organization. Upper chart characterizes the expression state and coding potential of ‘Honeycrisp’ novel transcripts. Lower chart characterizes the genomic organization of ‘Honeycrisp’ novel transcripts. (C) Assignment of BLASTn matches of ‘Honeycrisp’ novel transcripts by genus.

The recent availability of a high-quality apple genome sequence and exhaustive depth of our transcriptional data provided the opportunity to document genes expressed in the apple bourse shoot apex with high-resolution and accuracy. Our reference-based transcriptome assembly cataloged a total of 104,690 transcripts arising from 58,452 loci (Table 1 and S1 File). This extends considerably the previously annotated gene content of the GDDH13 genome, which was based on nine RNA-seq libraries representing diverse structures, including the shoot apex, along with cDNAs and expressed sequence tags (ESTs) cataloged in NCBI databases. Our sequence and assembly results complemented the reference annotation with the identification of an additional 11,264 novel transcriptional models and 39,227 ’Honeycrisp’-specific isoforms of annotated transcripts (~10.8% and ~37.5% of the assembled transcripts, respectively; Fig 2A). These novel transcripts comprised 23,034 novel exons and originated from 8,753 previously unidentified loci. We further characterized these novel transcripts in terms of length, expression level, coding potential, genomic organization, and homology with known, expressed genes (Fig 2B and S2–S4 Tables).

**Table 1:** Sequencing and transcriptome assembly statistics.

Measured Statistic	Value
Bases Sequenced	39,201,481,600
Total Sequence Reads	392,014,816
Mean Overall Read Mapping Rate	90.10%
Total Number of Transcript Models	104,690
Total Number of Genes	58,452
Novel Exons	23,034
Novel Introns	11,152
Novel Loci	8,753
Average Transcripts per Loci	3.4
Average Transcript Length (bases)	1,617

The majority of these transcripts (81.2%; 9,096) were expressed (FPKM > 1; TPM 1.19–1.72; Fig 2B and S3 Table). Of those expressed transcripts, 63.7% were predicted to encode proteins. About 46% of the expressed-coding transcripts were located in previously annotated intergenic regions. The remaining 56% showed some positional overlap with previously annotated genes (Fig 2B). In total, 73.8% (8,310) of the novel transcripts showed significant (E-value < 1e^-10^) nucleotide sequence homology to previously cataloged, expressed genes from *Malus spp*. (Fig 2C). These genes included 163 distinct loci encoding the *M*. *floribunda HcrVf*-like and *M*. x *domestica Rvi15* apple scab (*Venturia inaequalis*) resistance genes, and 159 loci encoding the *M*. x *robusta* fire blight (*Erwinia amylovora*) resistance genes. A total of 1,304 reference-mapped transcripts exhibited no significant homology to any sequence cataloged in the NCBI nt database (S2 Table).

Reads that did not align with the reference genome may represent sequence from uncharted segments of the apple genome including extrachromosomal DNAs or loci that are extremely diverged between GDDH13 and ’Honeycrisp’, or may be derived from exogenous biota. We assembled unmapped reads *de novo* into contiguous sequences (S2 and S3 Files) (see *Methods*), and evaluated the potential of the contigs to represent authentic apple transcripts. A total of 5,542 potential transcripts, representing 4,737 gene models, showed apparent expression values >100 FPKM in at least three of the sequencing libraries. About 39% of this subset of *de novo* transcripts were predicted to encode proteins (S5 Table and S1A Fig). About 75% of the 5,542 strongly expressed potential transcripts displayed significant homology to cataloged *Malus* sequences, and another 15% to sequences from related Rosaceae genera (S5 Table and S1B Fig).

### Identification of flowering gene homologs

Although genes with anticipated roles in flowering have previously been identified in apple, there has often been confusion and conflicting reports regarding gene identity, copy number, and expression pattern. This is most likely due to the existence of closely related orthologs for some of these genes, the heterogeneous and paleo-allopolyploid nature of the apple genome, and the inability of some previous approaches to discriminate among closely related sequences. The ~40 billion bases of transcriptional sequence data from the shoot apex analyzed in this project, as well as our identification of novel genes, provided the opportunity to resolve gene identities and estimate orthologous relationships. We identified a set of 180 Arabidopsis genes with flowering-related annotations (see *Methods*), and searched the combined GDDH13 / ’Honeycrisp’ transcriptome for expressed sequences with significant homology (E-value < 1e^-12^). In each case, the open reading frame translation from the primary designated transcript of the Arabidopsis gene was used to query the primary translations from both the annotated and novel reference-based transcriptional models, as well as the ORF-containing *de novo* transcriptional models. The highest-scoring, matching sequences were then used reciprocally to query a comprehensive database of open reading frame translations from Arabidopsis. Using this approach, we identified a total of 321 apple counterparts to 125 Arabidopsis genes. For further discussion, we refer to this collection as ’flowering gene homologs’. Three of the identified apple genes had not previously been annotated in the GDDH13 reference genome (S9 Table).

**Fig 3:**
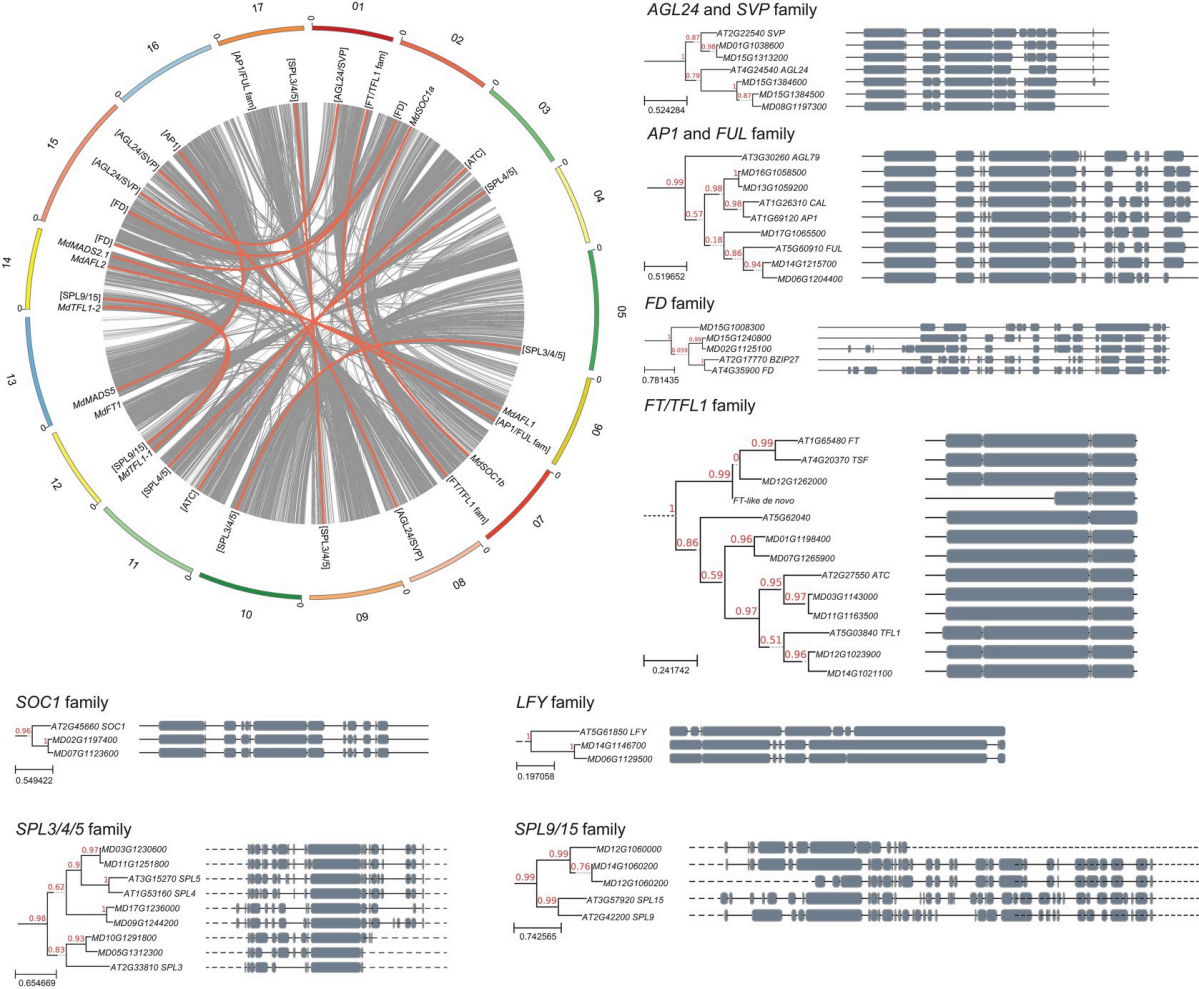
Analysis of genomic collinearity and phylogeny for 15 flowering gene families. The Circos plot at upper left depicts the chromosomal positions of flowering genes in the apple genome and their collinear relationships (red links). The background grey links represent the genomic collinearity within the apple genome. Chromosomes with significant homeology are depicted using similar colors. Phylogenetic trees are shown for each gene or gene family. For the phylogenetic trees, protein structure is depicted to the right of each gene, with conserved domains shown as grey blocks.

At least 106 of the 125 Arabidopsis flowering genes had multiple homologs. Previous research indicates that genes in apple generally exist as duplicates as a result of an ancient whole-genome duplication [56]. We analyzed genomic synteny for all of the 125 flowering gene families (Fig 3 and S9 Table). Based on chromosomal positions, a simple genome duplication appears to have contributed to family expansion for at least 86 of these 106 Arabidopsis genes, and tandem duplication contributed to expansion for at least 14 (S10 Table).

We identified 55 Arabidopsis flowering-related genes lacking a reciprocal homolog in the combined reference shoot apex transcriptome. These unrepresented genes included several functioning in the Arabidopsis vernalization-response pathway, including *FLOWERING LOCUS C* (*FLC*) and its sibling *MADS AFFECTING FLOWERING* (*MAF*), *FRIGIDA* (*FRI*), and *VERNALIZATION INSENSITIVE 3* (*VIN3*). This result is consistent with the apparently cold-independent initiation of flowers during the summer period in apple [57]. The gene previously described as an *FLC* homolog (MD09G1009100) by Takeuchi et al. [58] and Nishiyama et al. [59] was found to be not closely related to *FLC* in our study (S2D Fig). Other Arabidopsis flowering genes without clear apple representatives included 16 additional members of the *AGAMOUS-*like (*AGL*) MADS-box gene superfamily. These results are consistent with the observed rapid evolution and diversification of the large MADS-box genes observed in apple and other plants [60, 61].

We focused further study on a subset of flowering genes that have been intensively studied both in Arabidopsis and other plants, as previously reviewed [62]. Apart from *FLC*, this subset included *AGL24*, *AP1*, *FD*, *FUL*, *FT*, *LFY*, *SOC1*, *SPL3*, *SPL4*, *SPL5*, *SPL9*, *SPL15*, *SVP*, *TSF*, and *TFL1* (Table 2, Fig 3 and S2A–S2N Fig). In Arabidopsis, *FT* is transcribed in the leaves along with its paralog *TSF* and translocated to the apex [62]. In the apex, FT forms a complex with FD which activates transcription of *AP1* and *SPL3/4/5* directly. The FD/FT complex also indirectly activates the expression of *FUL* and *SOC1* [62]. In addition, *FUL* and *SOC1* expression is reinforced by *SPL9/15* [63]. This collective network promotes a phase change within the apex leading to floral initiation. *SOC1* and *AGL24* form a positive-feedback loop, promoting one another’s expression along with promoting *LFY* expression [64]. *LFY* expression is also directly promoted by *SPL3/4/5* and indirectly by *AP1* establishing floral meristem identity [63]. Negative regulators of this process are *SVP* and *TFL1*. *SVP* represses *FT* expression, whereas TFL1 competes with FT for complex formation with FD [62].

**Table 2:** Identified homologs of Arabidopsis flowering genes in apple.

Family			Locus	Clade	Alias	Citation
***AGL24*** ***SVP***	* *	* *	MD01G1038600	*AGL24/SVP*	* *	
	* *	* *	MD08G1197300	*AGL24/SVP*	* *	
	* *	* *	MD15G1313200	*AGL24/SVP*	* *	
	* *	* *	MD15G1384500	*AGL24/SVP*	* *	
	* *	* *	MD15G1384600	*AGL24/SVP*	* *	
***AP1*** ***CAL*** ***FUL*** ***AGL79***	***AP1*** ***CAL***	* *	MD13G1059200	*AP1*	*MdMADS5*	[16]
		* *	MD16G1058500	*AP1*	* *	
	* *	* *	MD06G1204400	*AP1/FUL fam*	* *	
	* *	* *	MD14G1215700	*AP1/FUL fam*	*MdMADS2*.*1*	[15]
	* *	* *	MD17G1065500	*AP1/FUL fam*	* *	
***FD*** ***BZIP27***	* *	* *	MD02G1125100	*FD*	* *	
	* *	* *	MD15G1008300	*FD*	* *	
	* *	* *	MD15G1240800	*FD*	* *	
***FT*** ***TSF*** ***AT5G62040 ATC*** ***TFL1***	***FT*** ***TSF***	* *	MD12G1262000	*FT*	*MdFT1*	[16]
		* *	FT-like *de novo*	*FT*	*MdFT2*	
	***TFL1*** ***ATC***	***ATC***	MD03G1143000	*ATC*	* *	
			MD11G1163500	*ATC*	* *	
		* *	MD12G1023900	*TFL1*	*MdTFL1-1*	[13, 14, 19]
		* *	MD14G1021100	*TFL1*	*MdTFL1-2*	
	* *	* *	MD01G1198400	*FT/TFL1 fam*	* *	
	* *	* *	MD07G1265900	*FT/TFL1 fam*	* *	
***LFY***	* *	* *	MD06G1129500	*LFY1*	*MdAFL1*	[11]
	* *	* *	MD14G1146700	*LFY2*	*MdAFL2*	
***SOC1***	* *	* *	MD02G1197400	*SOC1*	*MdSOC1a*	[16]
	* *	* *	MD07G1123600	*SOC1*	*MdSOC1b*	
***SPL3*** ***SPL4*** ***SPL5***	***SPL4*** ***SPL5***	* *	MD03G1230600	*SPL4/5*	* *	
		* *	MD11G1251800	*SPL4/5*	* *	
	* *	* *	MD05G1312300	*SPL3/4/5*	* *	
	* *	* *	MD09G1244200	*SPL3/4/5*	* *	
	* *	* *	MD10G1291800	*SPL3/4/5*	* *	
	* *	* *	MD17G1236000	*SPL3/4/5*	* *	
***SPL9*** ***SPL15***	* *	* *	MD12G1060000	*SPL9/15*	* *	
	* *	* *	MD12G1060200	*SPL9/15*	* *	
	* *	* *	MD14G1060200	*SPL9/15*	* *	

We reconstructed phylogenies for these genes, including the most homologous genes from both Arabidopsis and apple, and generated un-rooted trees (Fig 3). Apple genes related to *AGL24/SVP*, *AP1/FUL*, *FD*, *FT/TFL1*, *LFY*, *SOC1*, *SPL4/5*, and *SPL9/15* were included in well-defined (>90% bootstrap replicates) clades. The majority of these apple genes existed as pairs on homeologous chromosomes, as anticipated. Additional homologs likely resulted from tandem duplication, as evidenced by their close proximity (*e*.*g*., the *AGL24/SVP* clade pair *MD15G1384500*/*MD15G1384600*). Apple was previously found to contain two homologs of *FT*, one positioned on Chr. 4 (*MdFT2*) and the other on Chr. 12 (*MdFT1)* [16]. The GDDH13 genome contains only *MdFT1*. We assembled sequence reads that did not map to the GDDH13 genome (*see Methods*) and were able to identify a *MdFT2-*like transcript (*FT-like de novo*) (Table 2, Fig 3 and S2A–S2N Fig). This result suggests that the GDDH13 genome sequence is incomplete for Chr. 4 or that the GDDH13 doubled-haploid genotype lacks *MdFT2*. As anticipated from the reciprocal homology results (above), individual members of the *FLC/MAF* family showed no specific phylogenetic relationships with apple genes, although a group of three apple genes were often (88% of bootstrap replicates) placed into a clade with the *FLC/MAF* family (S2 Fig).

### Transcriptional analysis of the apple shoot apex during the floral transition

To gain insight into genetic pathway(s) associated with flowering in apple, we examined changes in gene expression occurring in the bourse shoot apex in the set of flowering-induced (thinned) trees spanning 2 DAFB to 70 DAFB. Because an appreciable fraction (~48%) of the apices did not initiate flowers during the year of this study (Fig 1), this set of genes represent those associated with vegetative apex activity (*i*.*e*. continued production of leaf primordia), as well as the transition to flowering. We identified a total of 12,661 reference-mapped genes, including ~100 flowering gene homologs, that exhibited significant changes in expression in at least one pairwise comparison among the five developmental stages evaluated.

**Fig 4:**
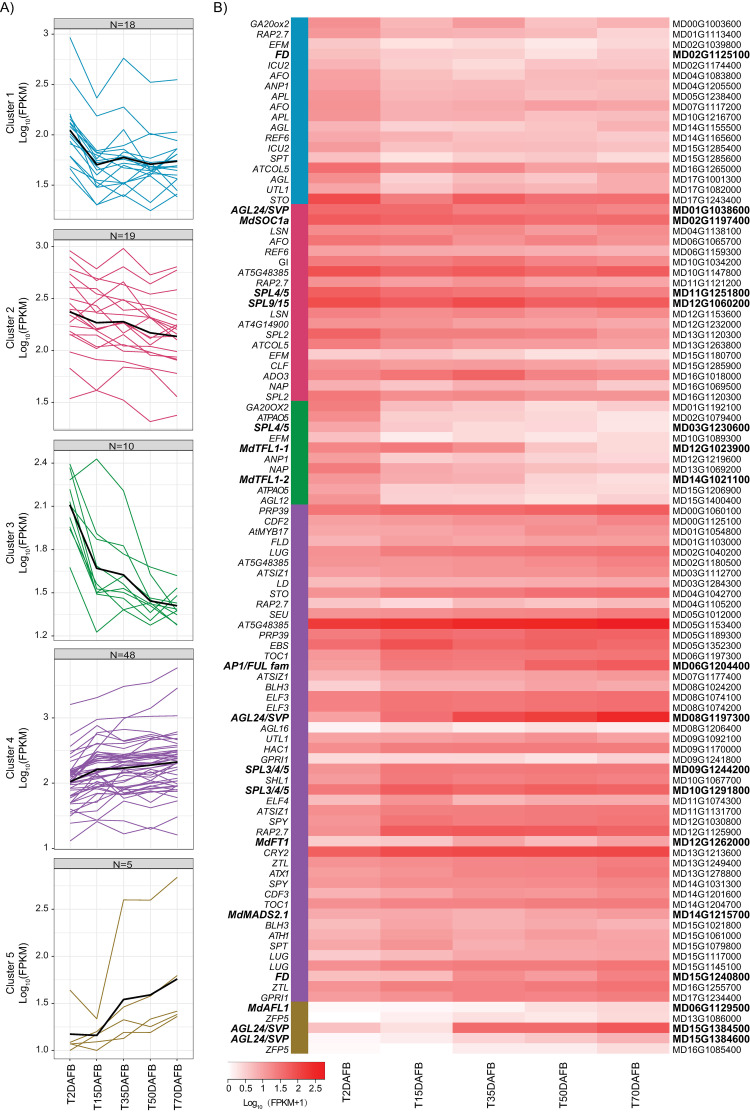
Expression patterns of flowering gene homologs in flowering-induced apices. A) Expression plots for flowering gene homologs that exhibited significant changes in expression across the study period. Expression profiles were grouped into similar patterns using *K*-means clustering (*k* = 5). For each cluster, the average expression pattern is represented by a black line. Expression levels (FPKM) were log_10_ transformed. N = number of flowering genes assigned to the respective cluster. B) Heatmap of expression values sorted by *K*-means cluster assignment. Homolog nomenclature is represented by gene symbols or Arabidopsis gene IDs on the left of the heatmap. On the right of the heatmap are the GDDH13 reference loci. Gene symbols and IDs shown in bold represent the subset of intensively studied flowering gene homologs listed in Table 2.

To define transcriptional programs potentially involving the ~100 flowering gene homologs, we clustered their expression profiles using a *K*-means approach (*k* = 5) (Fig 4). Clusters 1–3 represented genes that showed decreases in expression at some point during the period, consistent with a floral repressive role, or expression largely limited to vegetative phase. Cluster 1 genes (*n* = 18) generally showed a strong decrease in expression at the earliest studied interval, between 2 and 15 DAFB, with little or no expression change at later time points. This cluster included a homolog of *FD* (Fig 4). In contrast, Cluster 2 (*n* = 19) genes showed progressively decreasing expression over the course of the season. This cluster included *MdSOC1a*, as well as homologs of *AGL24/SVP*, *SPL4/5*, and *SPL9/15*. Most of the genes in Cluster 3 (*n* = 10) showed a strong decrease in expression at the earliest studied interval, between 2 and 15 DAFB, and continued decreasing expression at the later time points. This cluster included both *MdTFL1-1* and *MdTFL1-2*, although we noted that *MdTFL1-1* was upregulated between 2 and 15 DAFB (Fig 4). The strong decrease in expression of these two *TFL1* homologs during the anticipated period for floral initiation has previously been reported [13, 14, 16–19, 65, 66].

Genes in Clusters 4 and 5 showed generally increasing expression across the entire study period, suggesting promotive roles in flowering or expression domains linked with the floral phase. Cluster 4 genes (*n* = 48) showed steadily increasing expression across the period. These included *MdFT1*, as well as homologs of *AGL24/SVP*, *SPL3/4/5*, and *AP1/FUL*. Cluster 5 contained only five genes, and these were characterized by a generally more substantial increase in expression over the season. This cluster included *MdAFL1*, as well as a homolog of *AP1/FUL* and two homologs of *AGL24/SVP*. The increasing expression of *MdAFL1* and the *AP1/FUL* homolog reflects the increased expression of their counterparts during flowering in Arabidopsis. Expression of the two *AGL24/SVP* homologs was analogous with that of *AGL24* in Arabidopsis during the transition to a reproductive meristem [62].

### Transcriptional response to the presence of a fruit load

**Fig 5:**
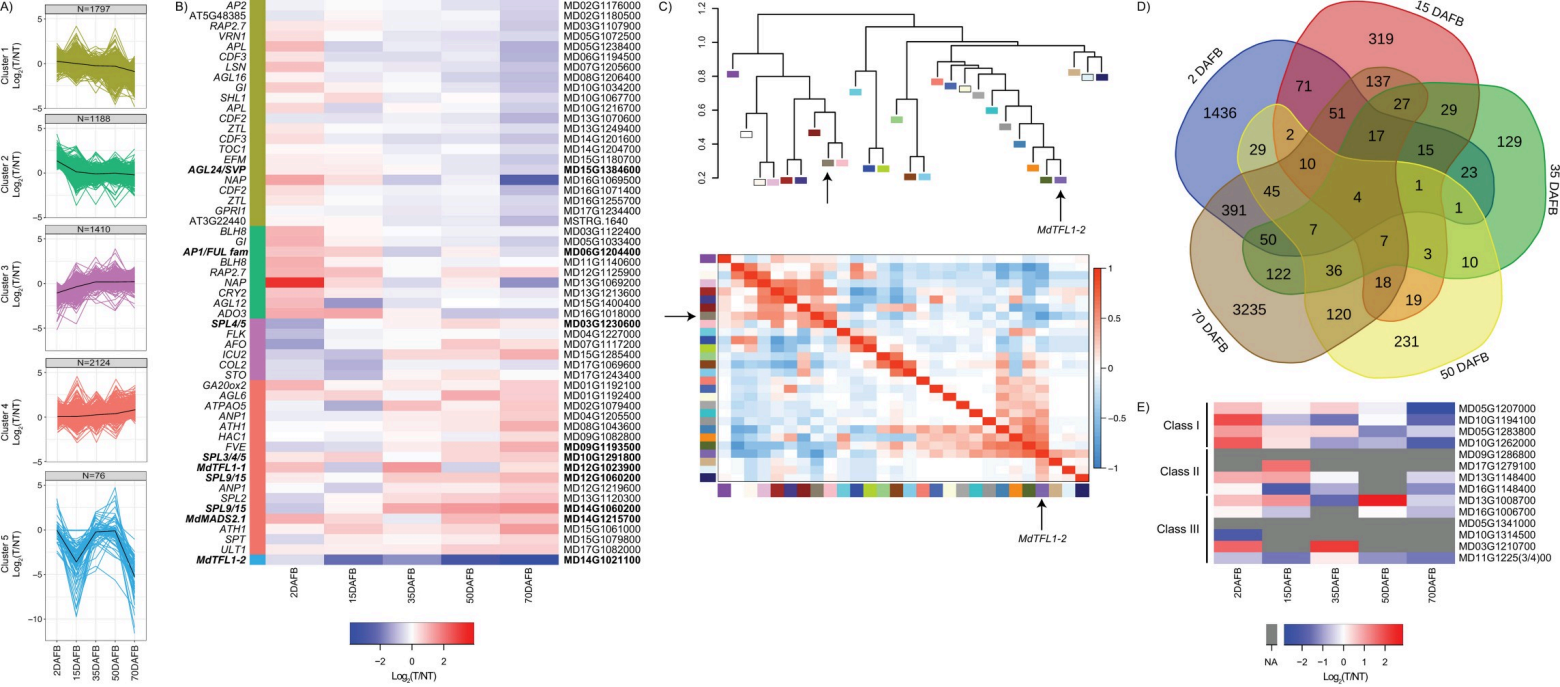
Expression characteristics of genes that are differentially expressed between thinned and non-thinned apices. A) Expression plots for flowering gene homologs that exhibited significant differences in expression between thinned (T) and non-thinned (NT) apices. Expression profiles were grouped into similar patterns using *K*-means clustering (*k* = 5). For each cluster, the average expression pattern is represented by a black line. Expression levels (FPKM) were log_10_ transformed. N = number of flowering genes assigned to the respective cluster. B) Heatmap of the fold difference in expression between thinned (T) and non-thinned (NT) samples for flowering gene homologs at each sample date. Homolog nomenclature is represented by gene symbols or Arabidopsis gene IDs on the left of the heatmap. On the right of the heatmap are the GDDH13 reference loci. Gene symbols and IDs shown in bold represent the subset of intensively studied flowering gene homologs listed in Table 2. C) Identification of genes co-expressed with *MdTFL1-2*. The dendrogram at top depicts the relationship among the modules. Colored rectangles represent all co-expression modules identified in this study. The module containing *MdTFL1-2* is shown in purple and is indicated. The module with the most significant negative correlation with the *MdTFL1-2* module is shown in brown and is indicated with a non-labeled arrow. The heat map at bottom represents the correlation values between modules (dark blue, strongly negatively correlated; dark red, strongly positively correlated). D) Venn diagram depicting the numbers of differentially expressed genes at each sample date. E) Heatmap of the fold difference in expression between thinned (T) and non-thinned (NT) samples for *GA2ox* homologs at each sample date. The GDDH13 reference locus id is shown at the right. The assigned class of each homolog is shown at the left. Cells for which no expression value was available are shown in gray (NA).

To identify genetic mechanisms that may be specifically involved in the repression of flowering by developing fruit, we compared gene expression between apices from the thinned (flowering-induced) and non-thinned (non-induced) trees at each time point over the study. At the 15 and 35 DAFB sampling times, fruit had reached ~10 mm and ~20 mm in diameter, respectively. At 50 DAFB, fruit had reached ~ 30 mm in diameter, and at 70 DAFB, fruit was ~40 mm in diameter. At 70 DAFB, seeds and embryos were still immature, but had reached their final size. Fruit and seed reached maturity at ~120–130 DAFB. We identified a total of 6,595 genes that were differentially expressed between the two conditions at one or more time points. Of these, 55 were included in the defined set of flowering gene homologs (Fig 5 and S11 Table).

*K*-means clustering identified five modal expression patterns. Genes in Cluster 1 were generally expressed to higher levels in non-thinned apices at later time points (50 and 70 DAFB) and thus could represent downstream floral repressors or genes expressed in the vegetative tissues of the apex. This expression pattern was exemplified by the *AGL24/SVP* homolog MD15G1384600 (Fig 4). This result suggests that the function of MD15G1384600 could be similar to *SVP* in maintaining vegetative identity [62].

Cluster 2 genes were generally expressed to higher levels in thinned apices at the earliest time points (2 and 15 DAFB) and could represent early flowering promoters. An example included in this cluster is the *AP1/FUL*-related gene, MD06G1204400. Genes in Clusters 3 and 4 showed generally increasing expression in thinned apices, relative to non-thinned apices, throughout the study period. Cluster 3 (higher expression in non-thinned apices only at the earliest time points) could represent early flowering repressors or genes expressed early in the vegetative tissues. This cluster contained the *SPL4/5* homolog, MD03G1230600. Cluster 4 (higher expression in thinned apices at the latest time point) might represent genes acting as promoters late in flowering, including floral development, or genes expressed in floral tissues. This cluster contained homologs of *SPL3/4/5* and *SPL9/15*, as well as the *AP1/FUL* homolog *MdMADS2*.*1* (Fig 5), which we had also found to increase in absolute expression over the season (Fig 4). Cluster 4 additionally included *MdTFL1-1*, which we had also found to show a strong decrease in absolute expression after 15 DAFB (Fig 4). This suggests that the presence of fruit promotes the seasonal decrease in expression of *MdTFL1-1*, as previously observed by Haberman et al. [18].

The final cluster, Cluster 5, contained a small group of genes (76) that showed greatly reduced expression in thinned apices, relative to non-thinned apices, at 15 DAFB and 70 DAFB (Fig 5). *MdTFL1-2* was the sole flowering gene homolog included in this group. Like *MdTFL1-1*, *MdTFL1-2* showed a decrease in expression throughout the season in flowering-induced apices, and the observed differential expression pattern suggests that the presence of fruit counteracts this seasonal decrease. This was also previously observed by Haberman et al. [18].

### *MdTFL1-2* expression profiling and identification of co-expressed genes

**Fig 6:**
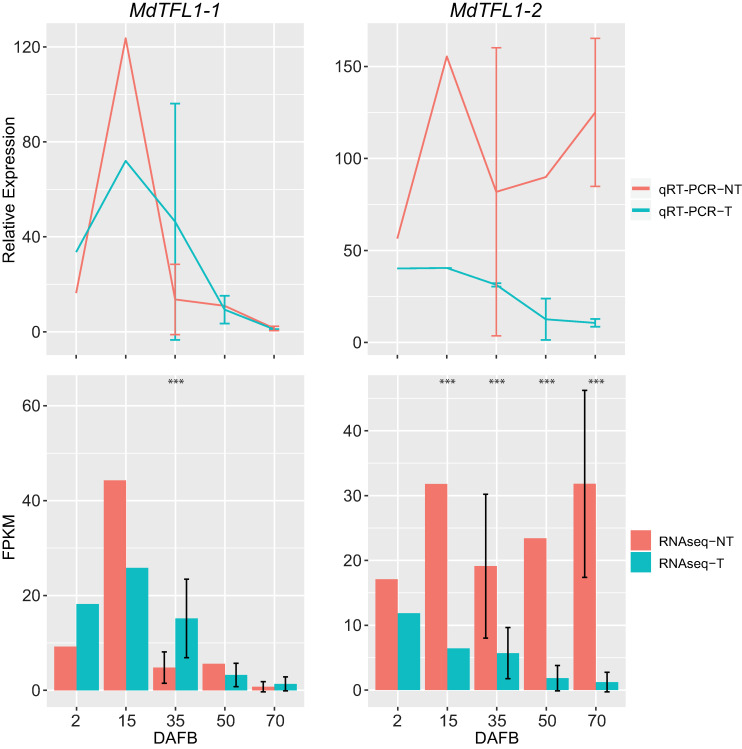
Expression profiles of *MdTFL1-1* and *MdTFL1-2* calculated from qPCR (upper panels) and RNAseq (lower panels). The correlation between the relative expression and FPKM values for *MdTFL1-1* and *MdTFL1-2* had R^2^ values of 0.97 and 0.99, respectively. Triple asterisks (***) indicate a q value <0.001. NT = non-thinned; T = thinned. Error bars represent the standard error between replicates.

This expression pattern of *MdTFL1-2* as reported previously by other groups, and here determined by RNA-seq, suggests this could be a key gene in regulating floral repression in the presence of fruit on the bourse shoot. We carried out qRT-PCR to quantify relative expression of both *MdTFL1-1* and *MdTFL1-2* to confirm the expression trend observed in the RNA-seq results. The results were generally consistent between the two approaches (Fig 6). Haberman et al. [18] previously reported that *MdTFL1-2* increased in expression between ~30 and ~60 DAFB in fruit bearing spurs. Our observations are distinct from those of Haberman et al. [18], as our results indicate that fruit promotes significant increased expression of *MdTFL1-2* as early as 15 DAFB. This early seasonal expression of *MdTFL1-2* overlaps with the period of floral induction/initiation in apple [4–7], and argues for a direct role for *MdTFL1-2* in repressing floral initiation, rather than a conceivable function in governing inflorescence architecture once initiation has occurred [54].

*MdTFL1-2* is expected to act in transcriptional regulation of flowering. Genes expressed similarly with *MdTFL1-2* (*i*.*e*. more strongly in non-thinned apices) could represent upstream promoters of *MdTFL1-2* expression or downstream positive targets. Considering only flowering gene homologs, in addition to the *AGL24/SVP* homolog MD15G1384600, these included homologs of *AGL16*, *BLH8*, *EFM*, *GPRI1*, *LSN*, and one homolog of *RAP2*.*7* (Fig 5). Conversely, genes expressed in a reciprocal manner to *MdTFL1-2* (*i*.*e*. less strongly in non-thinned apices), could represent upstream repressors of *MdTFL1-2* expression or downstream negative targets. This included homologs of *AGL6*, *MdMADS2*.*1*, *RAP2*.*7*, and various *SPLs* (Fig 5). In Arabidopsis, *AP1* represses *TFL1* expression, and this finding is consistent with a conserved function of *MdMADS2*.*1* in apple [67].

We also searched the subset of genes assigned to Cluster 5 for other potential upstream promoters or downstream regulatory targets of *MdTFL1-2* (Fig 5 and S12 Table). Of the 75 other genes assigned to this cluster, four would encode transcriptional regulator-like proteins. These included MD05G1203300, a homolog of *FERTILIZATION INDEPENDENT ENDOSPERM* (*FIE*). *FIE* encodes a component of a POLYCOMB REPRESSOR COMPLEX 2 (PRC2) protein that represses flowering and floral development in Arabidopsis [68]. In our study, this *FIE* homolog was expressed to higher levels in the non-thinned apices, relative to thinned, from 15 DAFB thru 70 DAFB (S4 File).

We employed a second, independent method to identify genes that could represent upstream promoters of *MdTFL1-2* or downstream targets through the identification of co-expression networks using the weighted gene correlation network analysis (WGCNA) approach [52]. This resolved 28 modules of co-expressed genes, with *MdTFL1-2* assigned to a module containing 200 genes (Fig 5C and S13 Table). This module contained homologs of *APL*, *EFM*, *SPL3*, and *ATH1* (S14 Table). We also identified a module with a strong negative correlation to *MdTFL1-2’s* module (Fig 5C and S15 Table). Here, we identified a total of 93 genes, including a distinct homolog of *ATH1* (S15 Table).

### Expression profiles of *GA2ox* and *GA20ox* genes

In our previous study of the mechanisms of the repression of flowering by GAs in apple, we found that *MdTFL1-2* was rapidly (within 2 days) upregulated in the shoot apex in response to exogenous GA_4+7_ [54]. In that study, we also found that exogenous GA resulted in the rapid upregulation of four genes classified as *GA2 OXIDASE* (*GA2ox*). Interestingly, all of the four *GA2ox* genes were included in the set of 6,595 genes differentially expressed in response to fruit load. A heat map of expression of these and additional *GA2ox* genes identified by Zhang et al. (2019) is shown in Fig 5E. The four *GA2ox* genes identified as differentially expressed shared a general pattern of higher expression in the thinned, relative to non-thinned, apices very early in the season (2 DAFB). Interestingly, as the season progressed, these genes showed higher expression in the non-thinned apices. This is consistent with previous studies by Guitton et al. [17] and Habermann et al. [18] showing that two of these four genes, MD05G1207000 and MD10G1194100, were expressed to higher levels in non-thinned samples at a similar sampling date (48 DAFB) as in our study. If cellular GA levels promote expression of these *GA2ox* genes, then the strong shift to higher expression in non-thinned apices could reflect increased GA levels in the apex, potentially driven by the presence of fruit.

In the previous study [54] we also documented that exogenous GA resulted in rapid downregulation of several genes encoding *GA20 OXIDASES* (*GA20ox*), which participate in GA biosynthesis and are recognized to be subject to feedback repression in many contexts in various plants. Here, we observed that the *GA20ox* homolog MD01G1192100 was expressed to relatively higher levels in thinned apices at all time points (Fig 5B). Habermann et al. [18] also reported higher expression of specific *GA20ox* homologs in thinned apices, including MD01G1192100. Thus, this observation might reflect lowered levels of bioactive GAs in thinned apices.

### Divergent expression patterns of homeologous gene pairs

**Fig 7:**
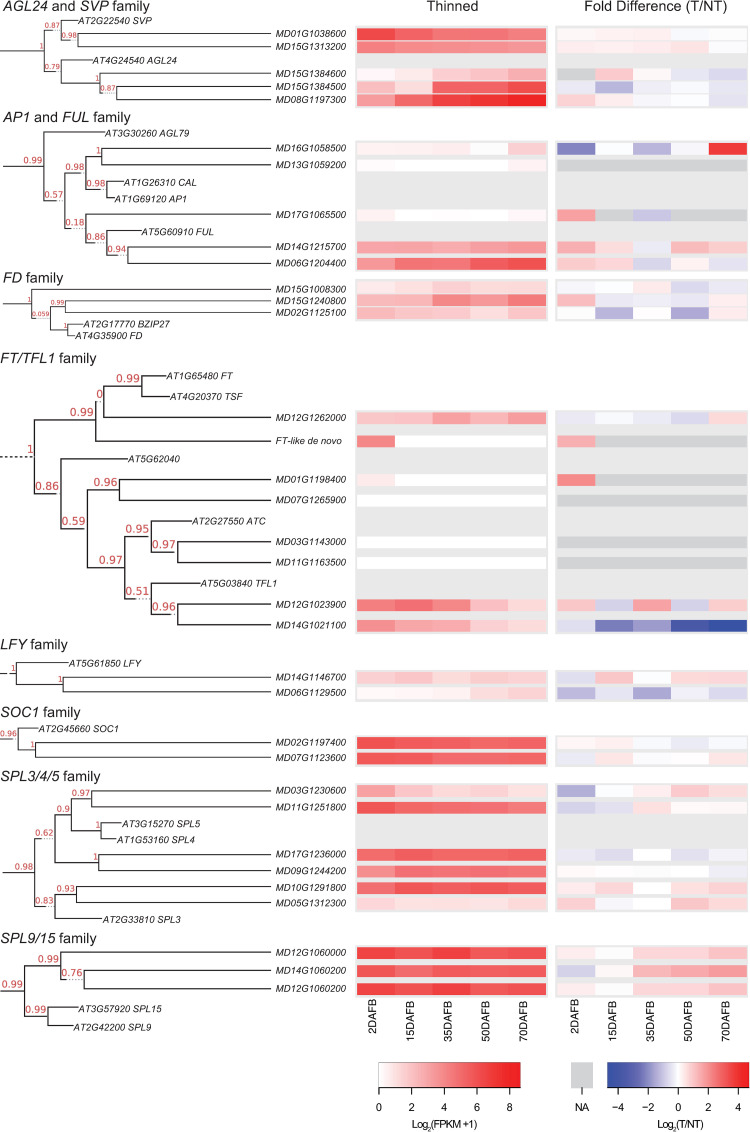
Phylogenetic trees and expression heatmaps for homeologous flowering genes. The phylogenetic trees are as shown in Fig 3. Heat maps represent expression at each sample date. In the left heatmap panel, expression values (FPKM +1) from thinned apices were log_2_ transformed. In the right heatmap panel, fold difference in expression between thinned (T) and non-thinned (NT) apices were log_2_ transformed. Cells for which no expression value was available are shown in dark gray (NA).

The differential regulation of the apple *TFL1-1* and *TFL1-2* genes is an interesting example of functional divergence of ancestrally related genes. Although expression of many of the key flowering gene homologs could not be reliably estimated, we identified several additional cases in which apparent gene duplication and/or gene family expansion was associated with distinctions in expression (Fig 7). For example, the *AP1/FUL* homeologs MD06G1204400 and MD14G1215700 showed distinct absolute expression patterns across the season, with MD06G1204400 increasing strikingly and MD14G1215700 remaining relatively constant. In contrast, the *SPL4/5* gene MD03G1230600 exhibited a strong decrease in expression as the season progressed, while expression of the homeologous MD11G1251800 stayed relatively constant. A third example was the *FD* homolog MD15G1230800, which was strongly increased at later time points, while its homeolog MD02G1125100 was not (Fig 7).

Expression of related genes was also differentially influenced by fruit load in several cases. Besides *TFL1-1/TFL1-2*, *MdAFL1* was more strongly expressed in the non-thinned apices at several time points, whereas *MdAFL2* was more weakly expressed. The distinctions in expression of these homologs of the intensively studied flowering genes underscores the importance of thoroughly indexing the genomic content and rigorously establishing phylogenetic relationships. Future characterization of function of these gene pairs can provide novel insight into the conserved and/or divergent genetic mechanism(s) that underlie their role in flowering in apple.

## Supporting information

S1 Fig Characterization of *de novo* assembled transcripts from reads that failed to map to the reference GDDH13 genome. A) Percentage of transcripts predicted to be coding or noncoding/ambiguous. B) Assignment of BLASTn matches of de novo assembled transcripts from unmapped reads by genus.
(DOCX)

S2 Fig Phylogenetic analysis and peptide sequence alignment for major flowering genes in Arabidopsis and apple. (DOCX)

S1 File Annotation file of assembled Honeycrisp transcript models from StringTie. (DOCX)

S2 File FASTA sequences of *de novo* transcripts assembled from unmapped reads. (DOCX)

S3 File Annotation file of *de novo* transcripts assembled from unmapped reads. (DOCX)

S4 File Differentially expressed genes. (DOCX)

S5 File Differentially expressed isoforms. (DOCX)

S1 Table Alignment metrics generated from RNA-SeQC for each individual read library that was mapped to the GDDH13 reference genome. DAFB = Days after full bloom, NT = Non-thinned, and T = Thinned.
(XLSX)

S2 Table Characterization of novel transcripts assembled from mapped reads to the reference genome. (XLSX)

S3 Table Novel transcripts that are expressed. (XLSX)

S4 Table Novel transcripts that are not expressed. (XLSX)

S5 Table Characterization of *de novo* transcripts assembled from unmapped reads. (XLSX)

S6 Table *De novo* transcripts that are expressed. (XLSX)

S7 Table *De novo* transcripts that are not expressed. (XLSX)

S8 Table Apple genes that were profiled using qRT-PCR and their respective primer and probe set sequence information. (XLSX)

S9 Table Identified homologs of Arabidopsis flowering genes in apple. Bold Gene IDs indicate gene families that underwent a phylogenetic analysis in addition to blast homolog identification. Underlined Gene IDs indicate loci identified in this paper that were not included in the reference annotation.
(XLSX)

S10 Table Homologs of flowering genes that share a collinear genomic organization. (XLSX)

S11 Table Genes that exhibited significant differential expression at one or more time points and are annotated as a flowering gene in response to fruit load. The q-value of the fold change is listed next to genes that exhibited a significant change in expression between the two conditions. Fold change was calculated as log_2_(T/NT).
(XLSX)

S12 Table Potential upstream promoters or downstream regulatory targets of *MdTFL1-2* that were identified in cluster 5. (XLSX)

S13 Table Apple genes and module color assignment determined by WCGNA. (XLSX)

S14 Table Apple genes co-expressed with *MdTFL1-2*. (XLSX)

S15 Table Apple genes with the strongest negative correlation with the module that contained *MdTFL1-2*. (XLSX)

## References

[1] Butler O. On the cause of alternate bearing in apple. Bulletin of the Torrey Botanical Club. 1917;44(2): 85–96.

[2] Jonkers H. Biennial bearing in apple and pear: a literature survey. Sci Hortic. 1979;11: 303–317.

[3] Monselise SP, Goldschmidt EE. Alternate bearing in fruit trees. In: Janick J, editor. Horticultural Reviews. Westport, CT: AVI Publishing Company Inc; 1982 pp.128–173.

[4] McArtney S, Hoover E, Hirst P, Booking IR. Seasonal variation in the onset and duration of flower development in ‘Royal Gala’ apple buds. J Hort Sci Biotech. 2001;76(5): 536–540.

[5] Foster T, Johnston R, Seleznyova A. A morphological and quantitative characterization of early floral development in Apple (Malus x domesitca Borhk.). Ann Bot. 2003;92: 199–206. 10.1093/aob/mcg120 12805080 PMC4243644

[6] Hoover E, De Silva N, McArtney S, Hirst P. Bud development and floral morphogenesis in four apple cultivars. J Hort Sci Biotech. 2004;79(6): 981–984.

[7] Dadpour MR, Movafeghi A, Grigorian W, Omidi Y. Determination of floral initiation in Malus domestica: A novel morphogenetic approach. Biol Plant. 2011;55(2): 243–252.

[8] Dennis FG, Neilsen JC. Physiological factors affecting biennial bearing in tree fruit: the role of seeds in apple. HortTechnology. 1999;9(3): 317–322.

[9] Luckwill LC, Weaver P, MacMillan J. Gibberellins and other growth hormones in apple seeds. J Hort Sci. 1969;44: 413–424.

[10] Wardlaw IF. The control of carbon partitioning in plants. New Phytol. 1990;116: 341–381. 10.1111/j.1469-8137.1990.tb00524.x 33874094

[11] Wada N, Cao Q, Kotoda N, Soejima J, Masuda T. Apple has two orthologues of FLORICAULA/LEAFY involved in flowering. Plant Mol Bio. 2002;49: 567–577. 10.1023/a:1015544207121 12081365

[12] Kotoda N, Wada M, Kusaba S, Kano-Murakami Y, Masuda T, Soejima J. Overexpression of MdMADS5, an APETALA1-like gene of apple, causes early flowering in transgenic Arabidopsis. Plant Sci. 2002;162(5): 679–687.

[13] Kotoda N, Wada M. MdTFL1, a TFL1-line gene of apple, retards the transition from the vegetative to reproductive phase in transgenic Arabidopsis. Plant Sci. 2005;168(1): 95–104.

[14] Hättasch C, Flachowsky H, Kapturska D, Hanke M. Isolation of flowering genes and seasonal changes in their transcript levels related to flower induction and initiation in apple (Malus domestica). Tree Physiol. 2008;28: 1459–1466. 10.1093/treephys/28.10.1459 18708327

[15] Cevik V, Ryder CD, Popovich A, Manning K, King Gj, Seymour GB. A FRUITFUL-like gene is associated with genetic variation for fruit flesh firmness in apple (Malus domestica Borkh.). Tree Genet Genomes. 2010;6: 271–279.

[16] Kotoda N, Hayashi H, Suzuki M, Igarashi M, Hatsuyama Y, Kidou S, et al Molecular characterization of FLOWERING LOCUS T-Like gene in apple (Malus x domestica Borkh.). Plant Cell Physiol. 2010;51(4): 561–575. 10.1093/pcp/pcq021 20189942

[17] Guitton B, Kelner JJ, Celton JM, Sabau X, Renou JP, Chagné D, et al Analysis of transcripts differentially expressed between fruited and deflowered ‘Gala’ adult trees: a contribution to biennial bearing understanding in apple. BMC Plant Biol. 2016;16: 55 10.1186/s12870-016-0739-y 26924309 PMC4770685

[18] Haberman A, Ackerman M, Crane O, Kelner JJ, Costes E, Samach A. Different flowering response to various fruit loads in apple cultivars correlates with degree of transcript reaccumulation of a TFL1-encoding gene. Plant J. 2016;87: 161–173. 10.1111/tpj.13190 27121325

[19] Mimida N, Kotoda N, Ueda T, Igarashi M, Hatsuyama Y, Iwanami H, et al Four TFL1/CEN-like genes on distinct linkage groups show different expression patterns to regulate vegetative and reproductive development in apple (Malus x domestica Borkh.). Plant Cell Physiol. 2009;50(2): 394–412. 10.1093/pcp/pcp001 19168455

[20] Kotoda N, Wada M, Komori S, Kidou S, Abe K, Masuda T, et al Expression pattern of homologues of floral meristem identity Genes LFY and AP1 during flower development in apple. J Amer Soc Hort Sci. 2000;125(4): 398–403.

[21] Li WM, Tao Y, Yao YX, Hao YJ, You CX. Ectopic over-expression of two apple Flowering Locus T homologues, MdFT1 and MdFT2, reduces juvenile phase in Arabidopsis. Biol Plant. 2010;54(4): 639–646.

[22] Tränkner C, Lehmann S, Hoenicka H, Hanke M, Fladung M, Lenhardt D, et al Over-expression of an FT-homologous gene of apple induces early flowering in annual and perennial plants. Planta. 2010;232(6): 1309–1324. 10.1007/s00425-010-1254-2 20811751

[23] Mimida N, Kidou S, Iwanami H, Moriya S, Abe K, Voogd C, et al Apple FLOWERING LOCUS T proteins interact with transcription factors implicated in cell growth and organ development. Tree Physiol. 2011;31(5): 555–566. 10.1093/treephys/tpr028 21571725

[24] Flachowsky H, Szankowski I, Waidmann S, Peil A, Tranker C, Hanke MV. The MdTFL1 gene of apple (Malus x domestica Borkh.) reduces vegetative growth and generation time. Tree Physiol. 2012;32: 1288–1301. 10.1093/treephys/tps080 23022687

[25] Munoz-Fambuena N, Mesejo C, Gonzalez-Max MC, Primo-Millo E, Agusti M, Iglesias DJ. Fruit regulates seasonal expression of flowering genes in alternate-bearing ’Moncada’ mandarin. Ann Bot. 2011;108: 511–519. 10.1093/aob/mcr164 21856639 PMC3158683

[26] Ziv D, Zviran T, Zezak O, Samach A, Irihimovitch V. Expression profiling of FLOWERING LOCUS T-like gene in alternate bearing ’Hass’ avocado trees suggests a role of PaFT in avocado flower induction. PloS One. 2014;9(10): e110613 10.1371/journal.pone.0110613 25330324 PMC4201567

[27] Kittikorn M, Okawa K, Ohara H, Kotoda N, Wada M, Yokoyama M, et al Effects of fruit load, shading, and 9,10-ketol-octadecadienoic acid (KODA) application on MdTFL1 and MdFT1 genes in apple buds. Plant Growth Regul. 2011;64: 75–81.

[28] Gasic K, Hernandez A, Korban SS. RNA extraction from different apple tissues rich in polyphenols and polysaccharides for cDNA library construction. Plant Mol Bio Rep. 2004;22: 437a–437g.

[29] Aronesty E. ea-utils: "Command-line tools for processing biological sequencing data"; https://github.com/ExpressionAnalysis/ea-utils 2011.

[30] Daccord N, Celton JM, Linsmith G, Becker C, Choisne N, Schijlen E, et al High-quality de-novo assembly of the apple genome and methylome dynamics of early fruit development. Nat Genet. 2017;49: 1099–1106. 10.1038/ng.3886 28581499

[31] Kim D, Langmead B, Salzberg SL. HISAT: a fast spliced aligner with low memory requirements. Nat Methods. 2015;12: 357–360. 10.1038/nmeth.3317 25751142 PMC4655817

[32] Pertea M, Pertea GM, Antonescu CM, Chang TC, Mendell JT, Salzberg SL. StringTie enables improved reconstruction of a transcriptome from RNA-seq reads. Nat Biotech. 2015;33: 290–295. 10.1038/nbt.3122 25690850 PMC4643835

[33] Pertea M, Kim D, Pertea GM, Leek JT, Salzberg SL. Transcript-level expression analysis of RNA-seq experiments with HISAT, StringTie and Ballgown. Nat Protoc. 2016;11: 1650–1667. 10.1038/nprot.2016.095 27560171 PMC5032908

[34] Trapnell C, Roberts A, Goff L, Pertea G, Kim D, Kelley DR, et al Differential gene and transcript expression analysis of RNA-seq experiments with TopHat and Cufflinks. Nat Protoc. 2012;7: 562–578. 10.1038/nprot.2012.016 22383036 PMC3334321

[35] Trapnell C, Williams B, Pertea G, Mortazavi A, Kwan G, van Baren J, et al Transcript assembly and quantification by RNA-Seq reveals unannotated transcripts and isoform switching during cell differentiation. Nat Biotech. 2010;28(5): 511–515. 10.1038/nbt.1621 20436464 PMC3146043

[36] Deluca DS, Levin JZ, Sivachenko A, Fennell T, Nazaire MD, Williams C, et al RNA-SeQC: RNA-seq metrics for quality control and process optimization. Bioinformatics. 2012;28(11): 1530–1532. 10.1093/bioinformatics/bts196 22539670 PMC3356847

[37] Pertea G, Pertea M. GFF Utilities: GffRead and GffCompare. F1000 Research. 2020;9: 304 10.12688/f1000research.23297.2 32489650 PMC7222033

[38] Yu-Jian K, De-Chang Y, Lei K, Mei H, Yu-Qi M, Liping W, et al CPC2: a fast and accurate coding potential calculator based on sequence intrinsic features. Nucleic Acids Res. 2017;45(W1): W12–W16. 10.1093/nar/gkx428 28521017 PMC5793834

[39] Li A, Zhang J, Zhou Z. PLEK: a tool for predicting long non-coding RNAs and messenger RNAs based on an improved k-mer scheme. BMC bioinformatics. 2014;15: 311 10.1186/1471-2105-15-311 25239089 PMC4177586

[40] Wang L, Park HJ, Dasari S, Wang S, Kocher JP, Li W. CPAT: Coding-potential assessment tool using an alignment-free logistic regression model. Nucleic Acids Res. 2013;41(6): e74 10.1093/nar/gkt006 23335781 PMC3616698

[41] Haas BJ, Papanicolaou A, Yassour M, Grabherr M, Blood PD, Bowden J, et al De novo transcript sequence reconstruction from RNA-seq using the Trinity platform for reference generation and analysis. Nat Protoc. 2013;8(8): 1494–1512. 10.1038/nprot.2013.084 23845962 PMC3875132

[42] Berardini TZ, Reiser L, Li D, Mezheritsky Y, Muller R, Strait E, et al The Arabidopsis Information Resource: Making and mining the “gold standard” annotated reference plant genome. Genesis. 2015;53: 474–485. 10.1002/dvg.22877 26201819 PMC4545719

[43] Camacho C, Coulouris G, Avagyan V, Ma N, Papadopoulos J, Bealer K, et al BLAST+: architecture and applications. BMC Bioinformatics 2008; 10:421. 10.1186/1471-2105-10-421 PMC2803857 20003500

[44] Geer LY, Marchler-Bauer A, Geer RC, Han L, He J, He S, et al The NCBI BioSystems database. Nucleic Acids Res. 2010;38: D492–D496. 10.1093/nar/gkp858 19854944 PMC2808896

[45] Huerta-Cepas J, Serra F, Bork P. ETE 3: Reconstruction, analysis and visualization of phylogenomic data. Mol Biol Evol. 2016;33(6): 1635–1638. 10.1093/molbev/msw046 26921390 PMC4868116

[46] Sievers F, Wilm A, Dineen D, Gibson TJ, Karplus K, Li W, et al Fast, scalable generation of high-quality protein multiple sequence alignments using Clustal Omega. Mol Syst Biol. 2011;7: 539 10.1038/msb.2011.75 21988835 PMC3261699

[47] Price MN, Dehal PS, Arkin AP. FastTree 2—approximately maximum-likelihood trees for large alignments. PLoS One 2010;5(3): e9490 10.1371/journal.pone.0009490 20224823 PMC2835736

[48] Wang Y, Tang H, DeBarry JD, Tan X, Li J, Wang X, et al MCScanX: a toolkit for detection and evolutionary analysis of gene synteny and collinearity. Nucleic Acids Res. 2012;40(7): e49 10.1093/nar/gkr1293 22217600 PMC3326336

[49] Krzywinski MI, Schein JE, Birol I, Connors J, Gascoyne R, Horsman D, et al Circos: An information aesthetic for comparative genomics. Genome Res. 2009;19: 1639–1645. 10.1101/gr.092759.109 19541911 PMC2752132

[50] R Core Team. R: a language and environment for statistical computing. R Foundation for Statistical Computing, Vienna, Austria www.R-project.org. 2013.

[51] Goff L, Trapnell C, Kelley D. cummeRbund: Analysis, exploration, manipulation, and visualization of Cufflinks high-throughput sequencing data. 2013;R package version 2.22.0.

[52] Langfelder P, Horvath S. WGCNA: an R package for weighted correlation network analysis. BMC Bioinformatics. 2008;9: 559 10.1186/1471-2105-9-559 19114008 PMC2631488

[53] Zhang B, Horvath S. A General Framework for Weighted Gene Co-Expression Network Analysis. Stat Appl Genet Mol. 2005;4(1): 17 10.2202/1544-6115.1128 16646834

[54] Zhang S, Gottschalk C, van Nocker S. Genetic mechanisms in the repression of flowering by gibberellins in apple (Malus x domestica Borkh.). BMC Genomics. 2019; 20:747 10.1186/s12864-019-6090-6 31619173 PMC6796362

[55] Embree CG, Myra MTD, Nichols DS, Wright AH. Effect of blossom density and crop load on growth, fruit quality, and return bloom in ‘Honeycrisp’ apple. HortScience. 2007;42(7): 1622–1625.

[56] Velasco R, Zharkikh A, Affourtit J, Dhingra A, Cestaro A, Kalyanaraman A, et al The genome of the domesticated apple (Malus × domestica Borkh.). Nat Genet. 2010;42(10): 833–839. 10.1038/ng.654 20802477

[57] Peace CP, Bianco L, Troggio M, van de Weg E, Howard NP, Cornille A, et al Apple whole genome sequences: recent advances and new prospects. Hortic Res. 2019;6: 59 10.1038/s41438-019-0141-7 30962944 PMC6450873

[58] Takeuchi T, Matsushita MC, Nishiyama S, Yamane H, Banno K, Tao R. RNA-sequencing analysis identifies genes associated with chilling-mediated endodormancy release in apple. J Am Soc Hort Sci. 2018;143(3): 194–206.

[59] Nishiyama S, Matsushita MC, Yamane H, Honda C, Okada K, Tamada Y, et al Functional and expressional analysis of apple FLC-like in relation to dormancy progress and flower bud development. Tree Physiol. 2019;tpz111. 10.1093/treephys/tpz111 31728534

[60] Ng M, Yanofsky MF. Function and evolution of the plant MADS-box gene family. Nat Rev Genet. 2001;2: 186–195. 10.1038/35056041 11256070

[61] Tian Y, Dong Q, Ji Z, Chi F, Cong P, Zhou Z. Genome-wide identification and analysis of the MADS-box gene family in apple. Gene. 2015;555(2): 277–290. 10.1016/j.gene.2014.11.018 25447908

[62] Andrés F, Coupland G. The genetic basis of flowering responses to seasonal cues. Nat Rev Genet. 2012;13: 627–639. 10.1038/nrg3291 22898651

[63] Xu M, Hu T, Zhao J, Park M, Earley KW, et al Developmental functions of miR156-regulated SQUAMOSA PROMOTER BINDING PROTEIN-LIKE (SPL) genes in Arabidopsis thaliana. PLoS Genet. 2016;12(8): e1006263 10.1371/journal.pgen.1006263 27541584 PMC4991793

[64] Liu C, Chen H, Er HL, Soo HM, Kumar PP, et al Direct interaction of AGL24 and SOC1 integrates flowering signals in Arabidopsis. Development. 2008;135: 1481–1491. 10.1242/dev.020255 18339670

[65] Bradley D, Ratcliffe O, Vincent C, Carpenter R, Coen E. Inflorescence Commitment and Architecture in Arabidopsis. Science. 1997;275(5296): 80–83. 10.1126/science.275.5296.80 8974397

[66] Ratcliffe OJ, Bradley DJ, Coen ES. Separation of the shoot and floral identity in Arabidopsis. Development. 1999;126:1109–1120. 10021331 10.1242/dev.126.6.1109

[67] Goslin K, Zheng B, Serrano-Mislata A, Rae L, Ryan PT, Kwaśniewska K, et al Transcription factor interplay between LEAFY and APETALA/CAULIFLOWER during floral initiation. Plant Physiol. 2017;174(2):1097–1109. 10.1104/pp.17.00098 28385730 PMC5462026

[68] Kinoshita T, Harada JJ, Goldberg RB, Fischer RL. Polycomb repression of flowering during early plant development. PNAS. 2001;98(24):14156–14161. 10.1073/pnas.241507798 11698668 PMC61184

