# Supplementary material for: Genetic mechanisms associated with floral initiation and the repressive effect of fruit on flowering in apple (Malus x domestica Borkh)
Source: PLoS One. 2021 Feb 19;16(2):e0245487. doi: 10.1371/journal.pone.0245487 (PMC7894833; doi:10.1371/journal.pone.0245487)
Supplement: S1 Fig — A) Percentage of transcripts predicted to be coding or noncoding/ambiguous. B) Assignment of BLASTn matches of de novo assembled transcripts from unmapped reads by genus. (DOCX) [file pone.0245487.s001.docx]

**S1 Fig. Characterization of *de novo* assembled transcripts from reads that failed to map to the reference GDDH13 genome.** A) Percentage of transcripts predicted to be coding or noncoding/ambiguous. B) Assignment of BLASTn matches of *de novo* assembled transcripts from unmapped reads by genus.
