# Supplementary material for: Genetic mechanisms associated with floral initiation and the repressive effect of fruit on flowering in apple (Malus x domestica Borkh)
Source: PLoS One. 2021 Feb 19;16(2):e0245487. doi: 10.1371/journal.pone.0245487 (PMC7894833; doi:10.1371/journal.pone.0245487)
Supplement: S2 Fig — (DOCX) [file pone.0245487.s002.docx]

**S2 Fig. Phylogenetic analysis and sequence alignment for Arabidopsis and apple proteins most closely related to 16 Arabidopsis flowering gene families.** Distances between genes was determined using the Neighbor Joining method. Conserved domains are shown as grey blocks.


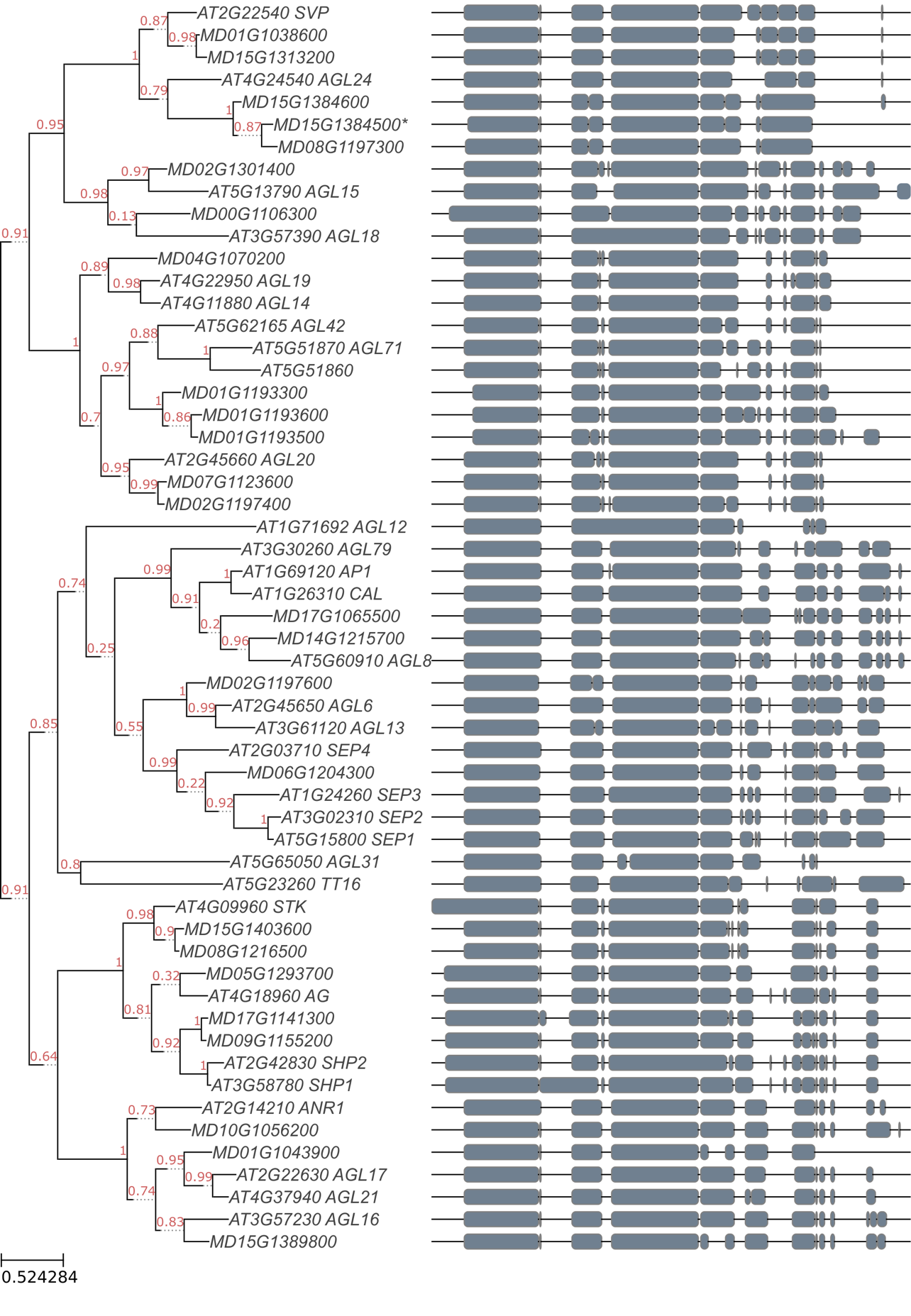


S2 Fig[a]. Phylogenetic analysis and sequence alignment for Arabidopsis and apple proteins most closely related to Arabidopsis *AGL24*. * Indicates reciprocal blast homolog.


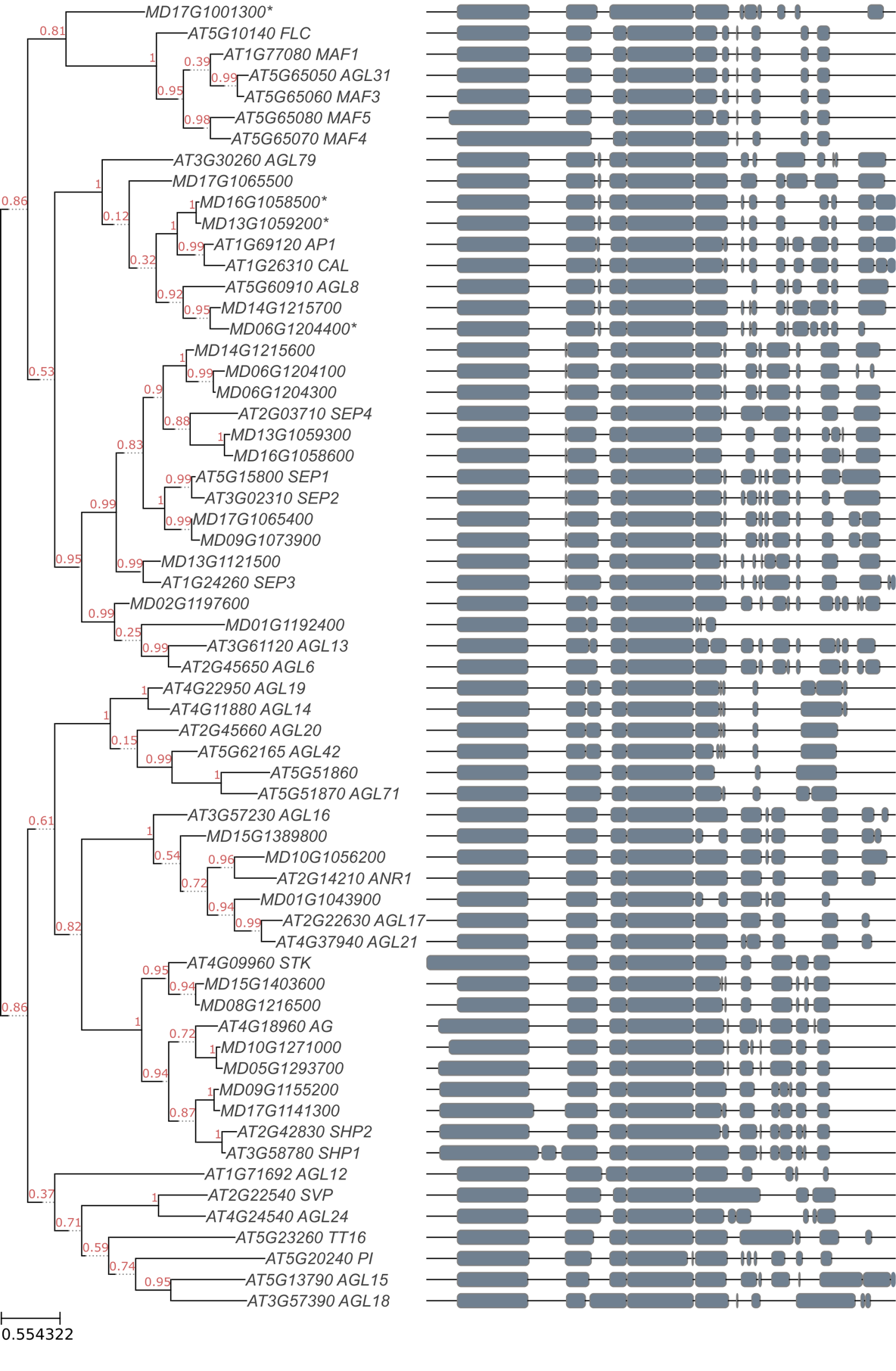


S2 Fig[b]. Phylogenetic analysis and sequence alignment for Arabidopsis and apple proteins most closely related to Arabidopsis *AP1*. * Indicates reciprocal blast homolog.


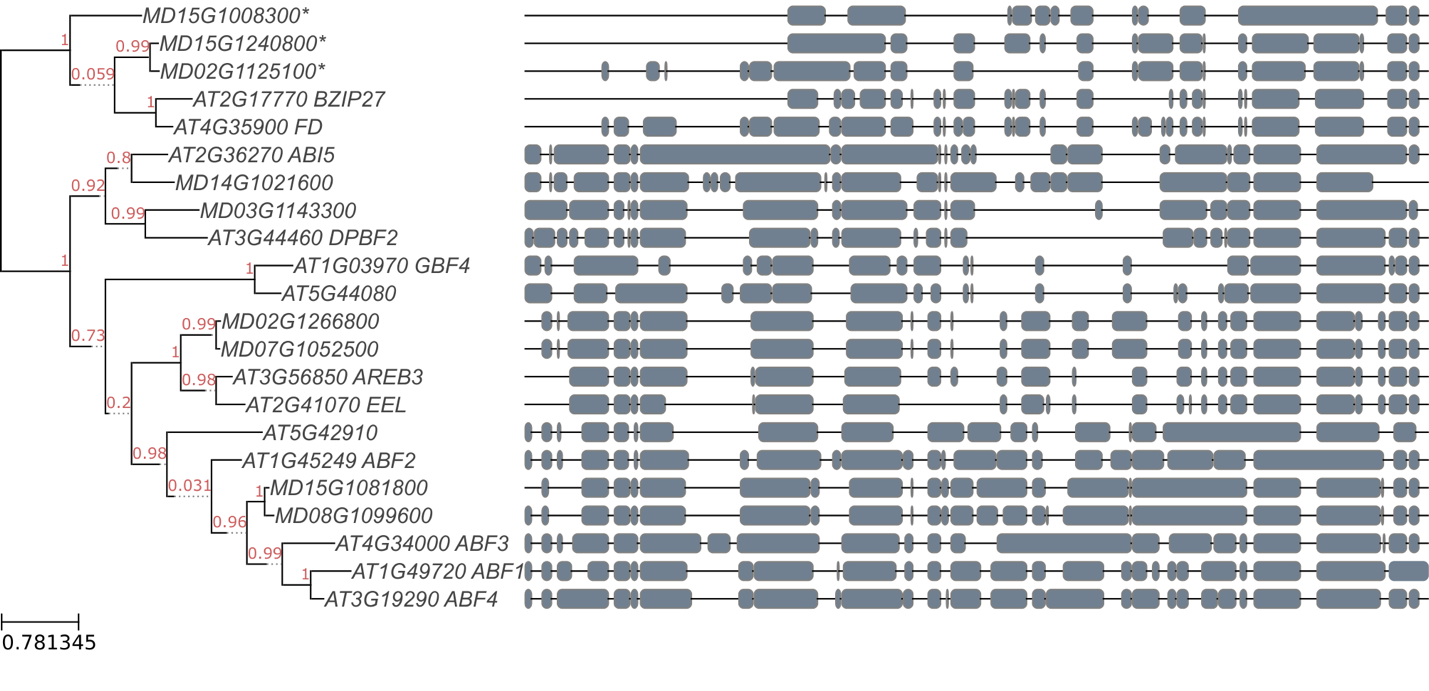


S2 Fig[c]. Phylogenetic analysis and sequence alignment for Arabidopsis and apple proteins most closely related to Arabidopsis *FD*. * Indicates reciprocal blast homolog.


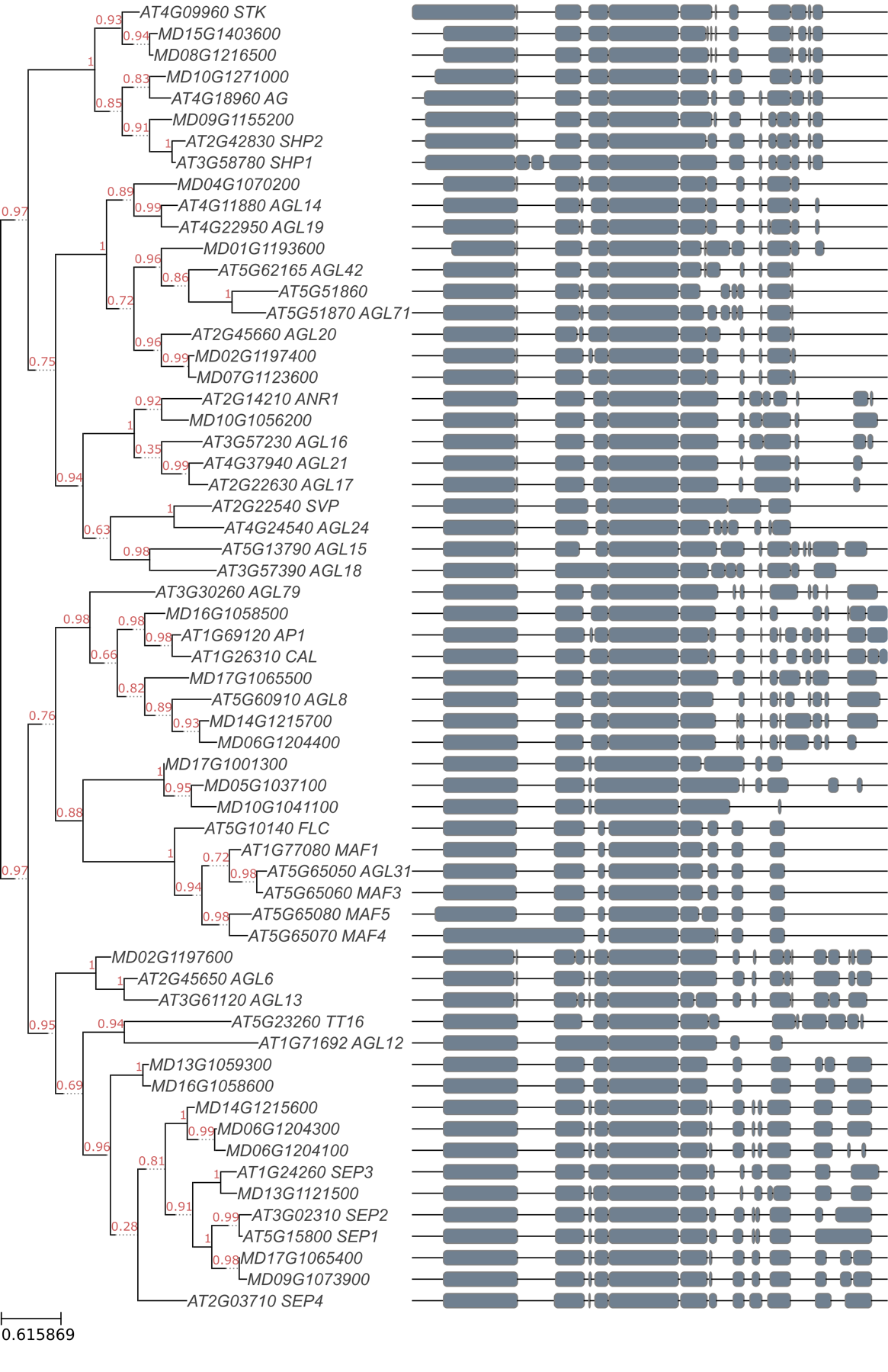


S2 Fig[d]. Phylogenetic analysis and sequence alignment for Arabidopsis *FLC* gene family and their apple counterparts. * Indicates reciprocal blast homolog.


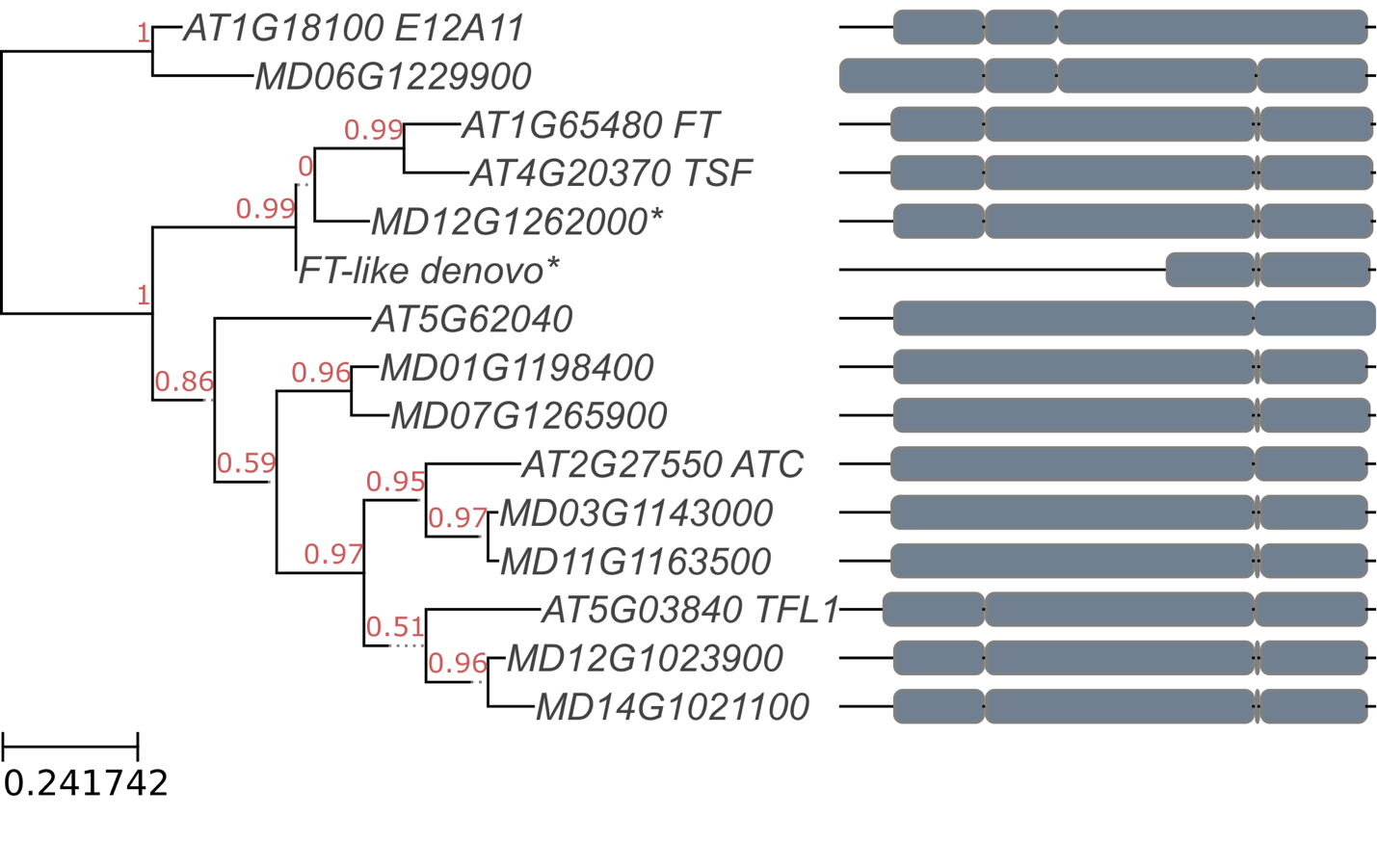


S2 Fig[e]. Phylogenetic analysis and sequence alignment for Arabidopsis and apple proteins most closely related to Arabidopsis *FT*. * Indicates reciprocal blast homolog.


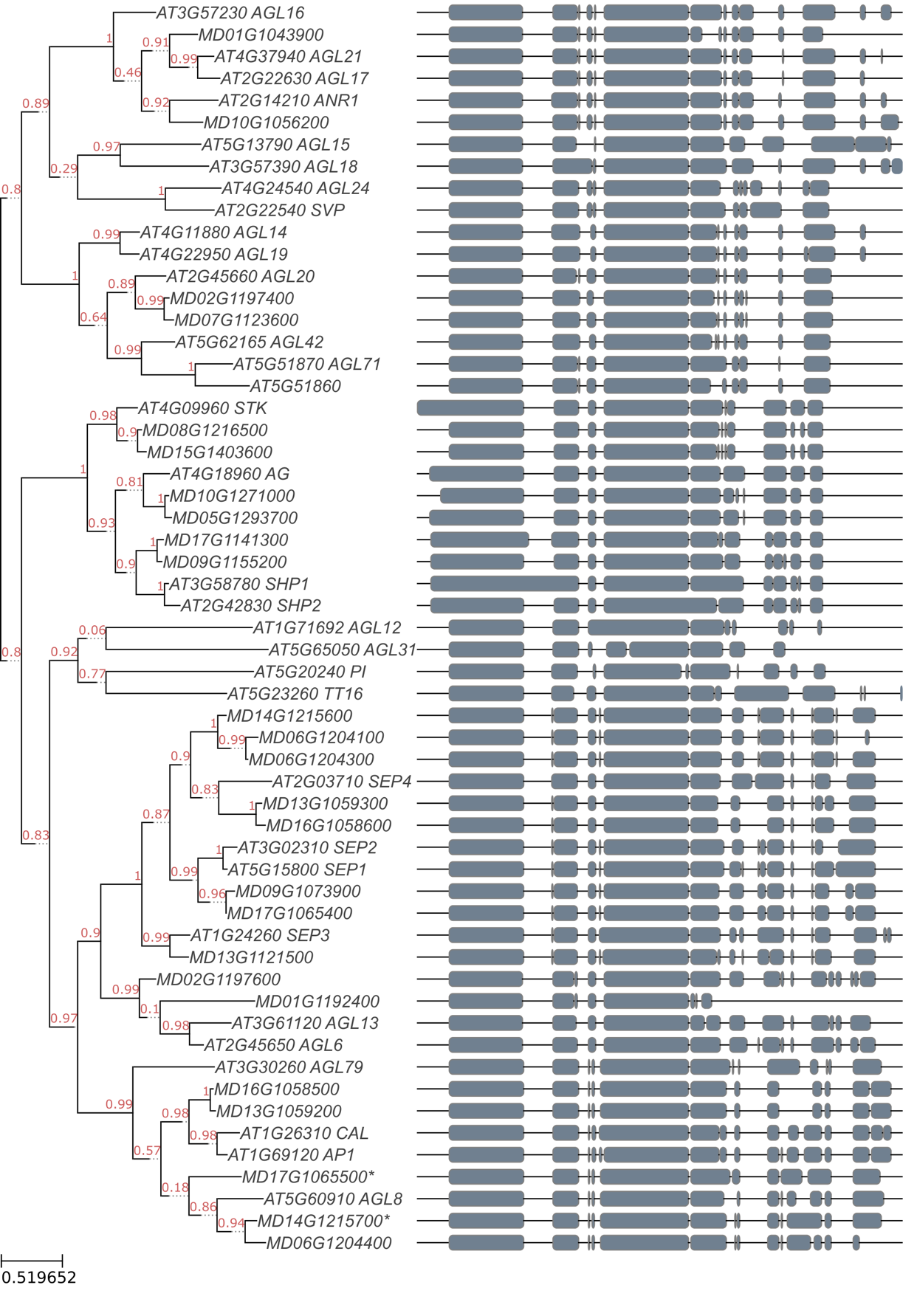


S2 Fig[f]. Phylogenetic analysis and sequence alignment for Arabidopsis and apple proteins most closely related to Arabidopsis *FUL*. * Indicates reciprocal blast homolog.


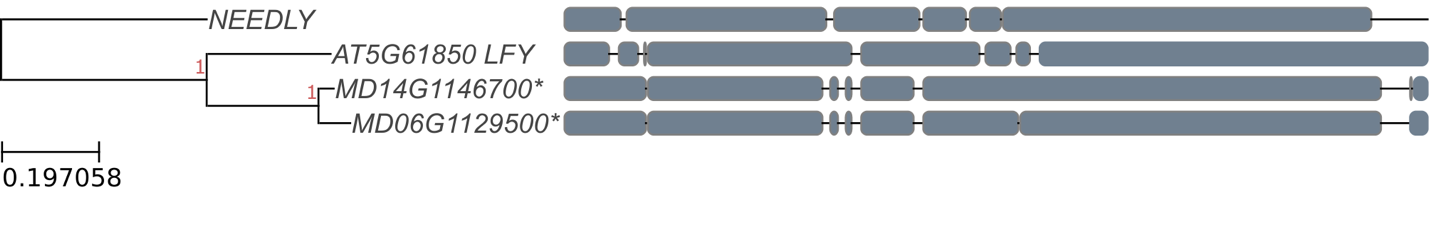


S2 Fig[g]. Phylogenetic analysis and sequence alignment for Arabidopsis and apple proteins most closely related to Arabidopsis *LFY*. The gene *NEEDLY,* a homolog of *LFY,* from *Pinus radiata* was used as an outgroup[Mouradov et al. 1998]. * Indicates reciprocal blast homolog.


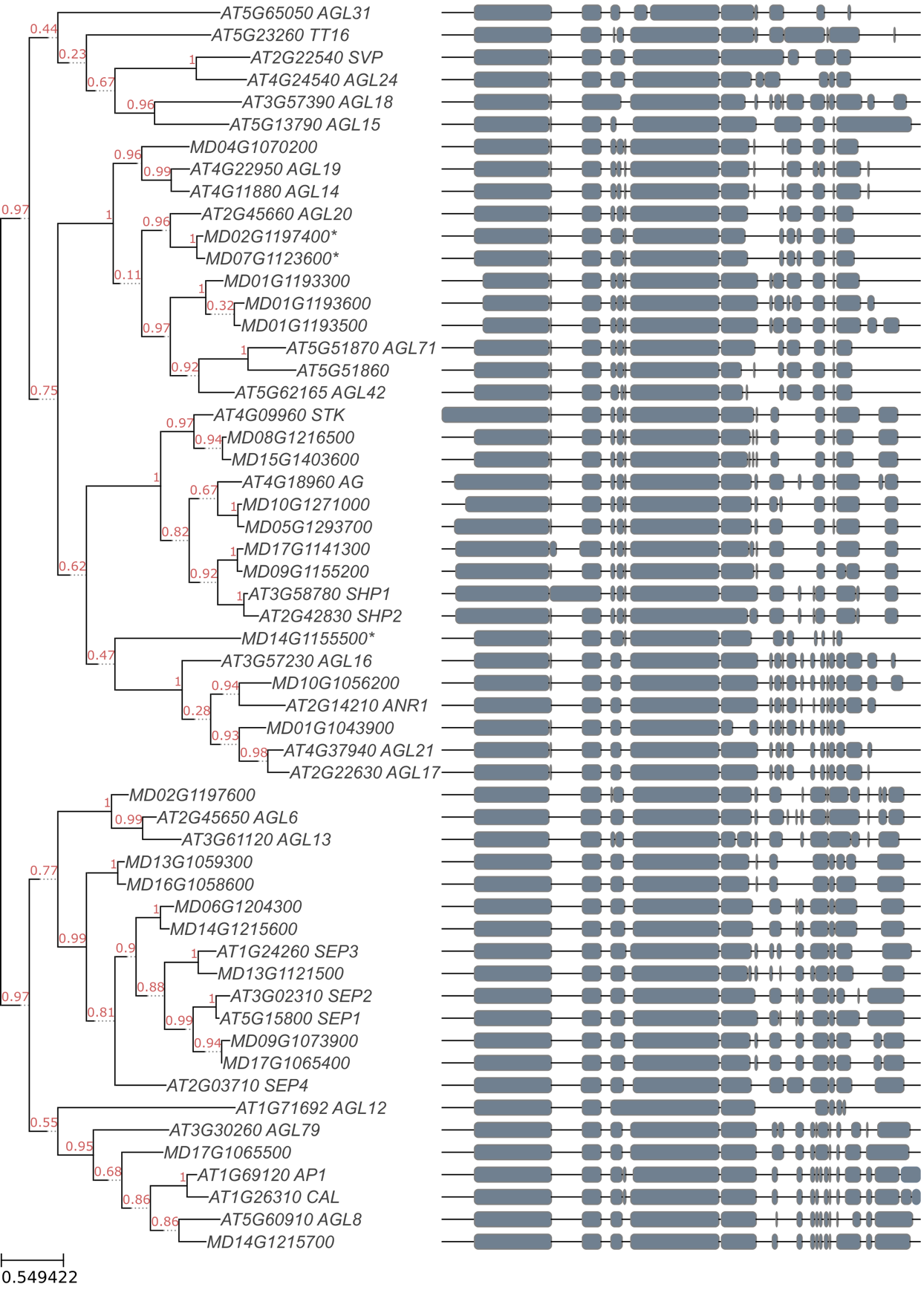


S2 Fig[h]. Phylogenetic analysis and sequence alignment for Arabidopsis and apple proteins most closely related to Arabidopsis *SOC1*. * Indicates reciprocal blast homolog.


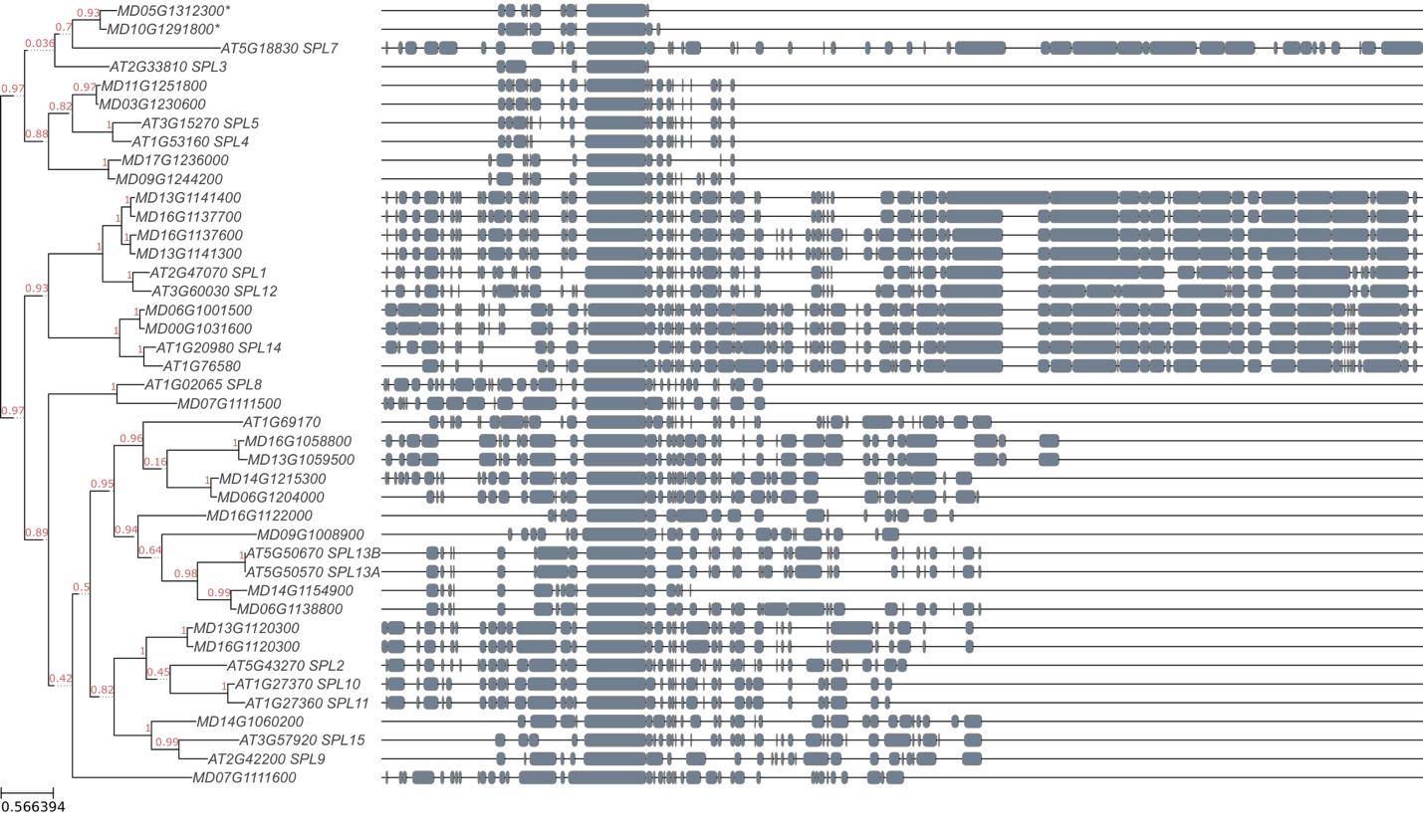


S2 Fig[i]. Phylogenetic analysis and sequence alignment for Arabidopsis and apple proteins most closely related to Arabidopsis *SPL3*. * Indicates reciprocal blast homolog.


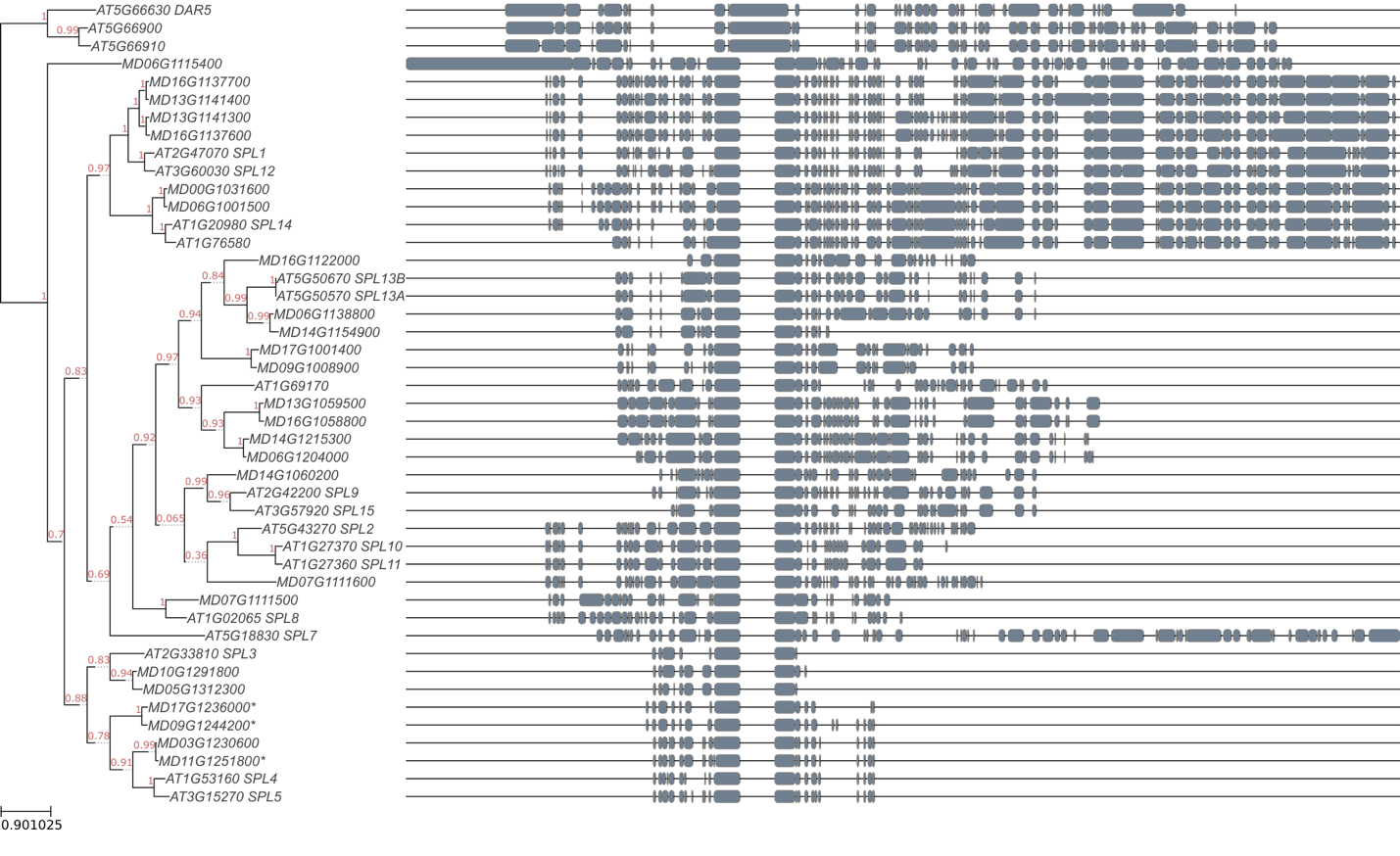


S2 Fig[j]. Phylogenetic analysis and sequence alignment for Arabidopsis and apple proteins most closely related to Arabidopsis *SPL4*. * Indicates reciprocal blast homolog.


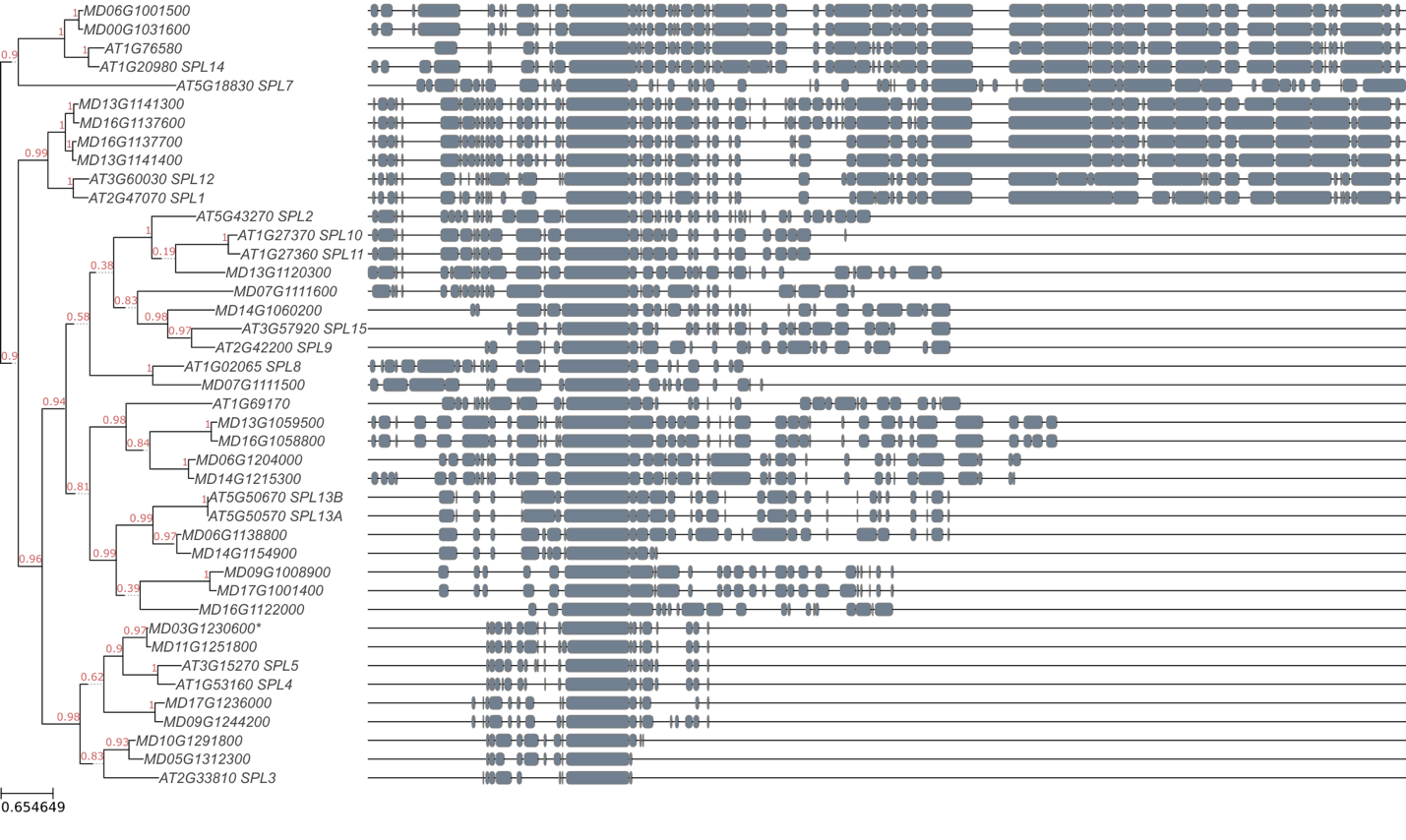


S2 Fig[k]. Phylogenetic analysis and sequence alignment for Arabidopsis and apple proteins most closely related to Arabidopsis *SPL5*. * Indicates reciprocal blast homolog.


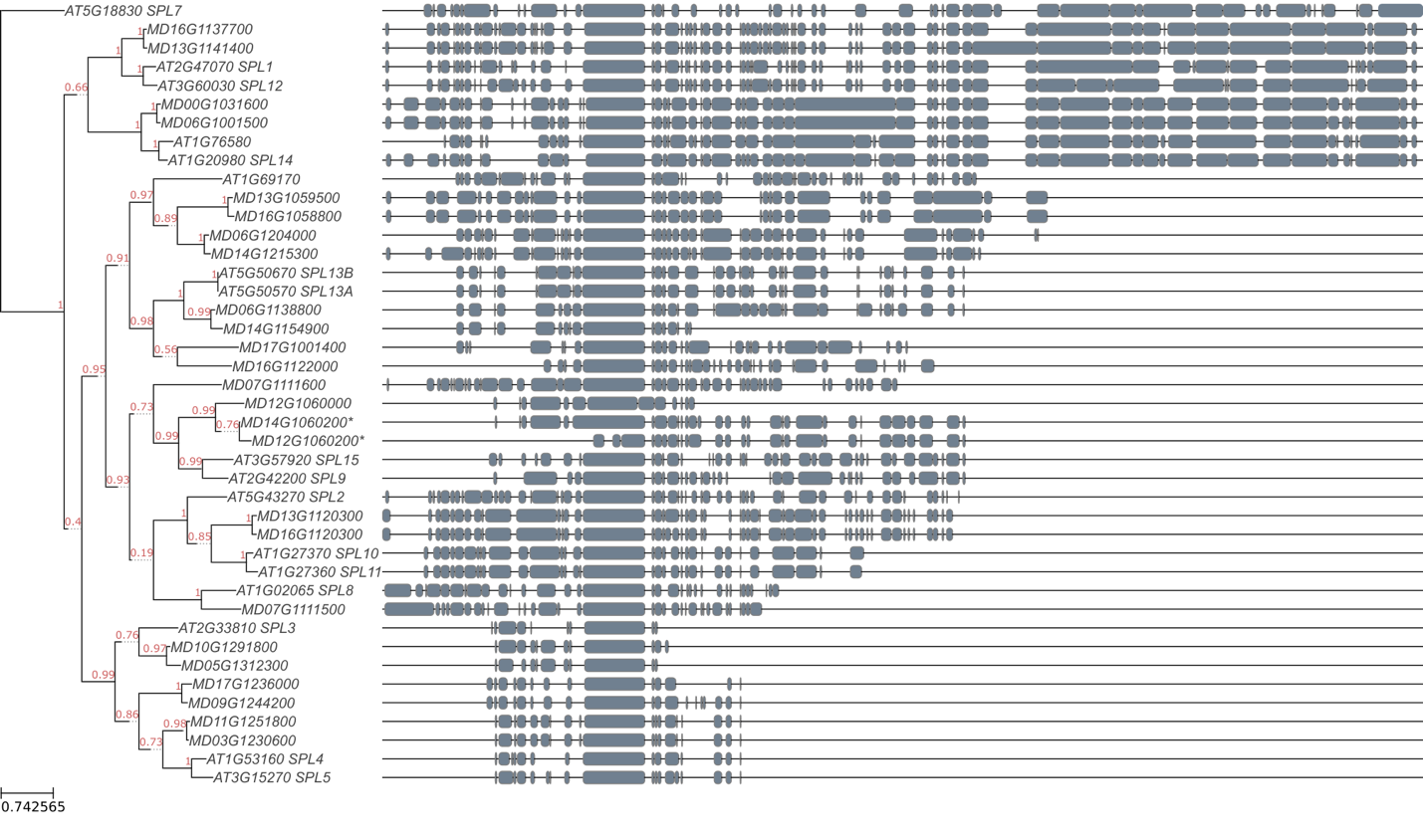


S2 Fig[l]. Phylogenetic analysis and sequence alignment for Arabidopsis and apple proteins most closely related to Arabidopsis *SPL9*. * Indicates reciprocal blast homolog.


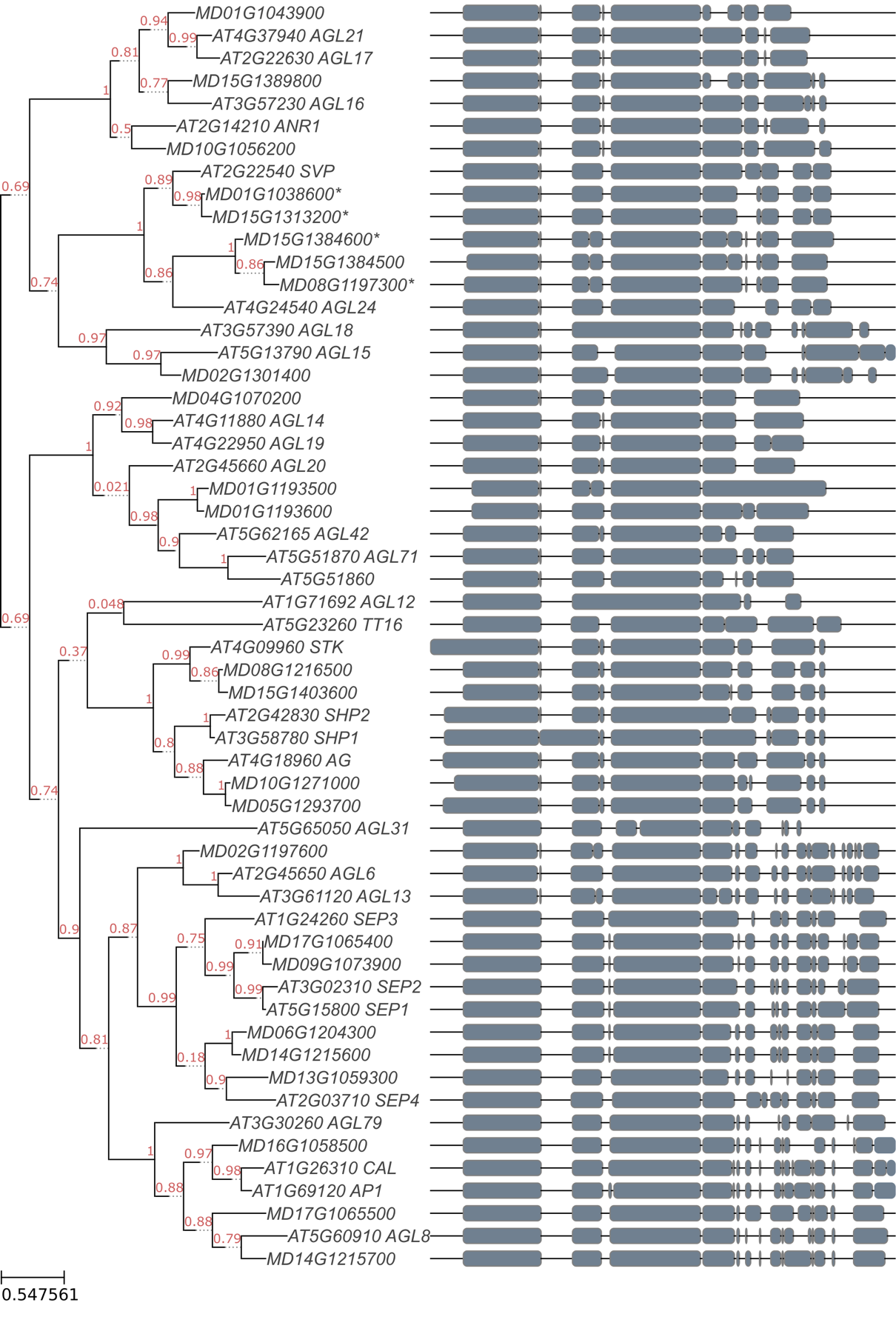


S2 Fig[m]. Phylogenetic analysis and sequence alignment for Arabidopsis and apple proteins most closely related to Arabidopsis *SVP*. * Indicates reciprocal blast homolog.


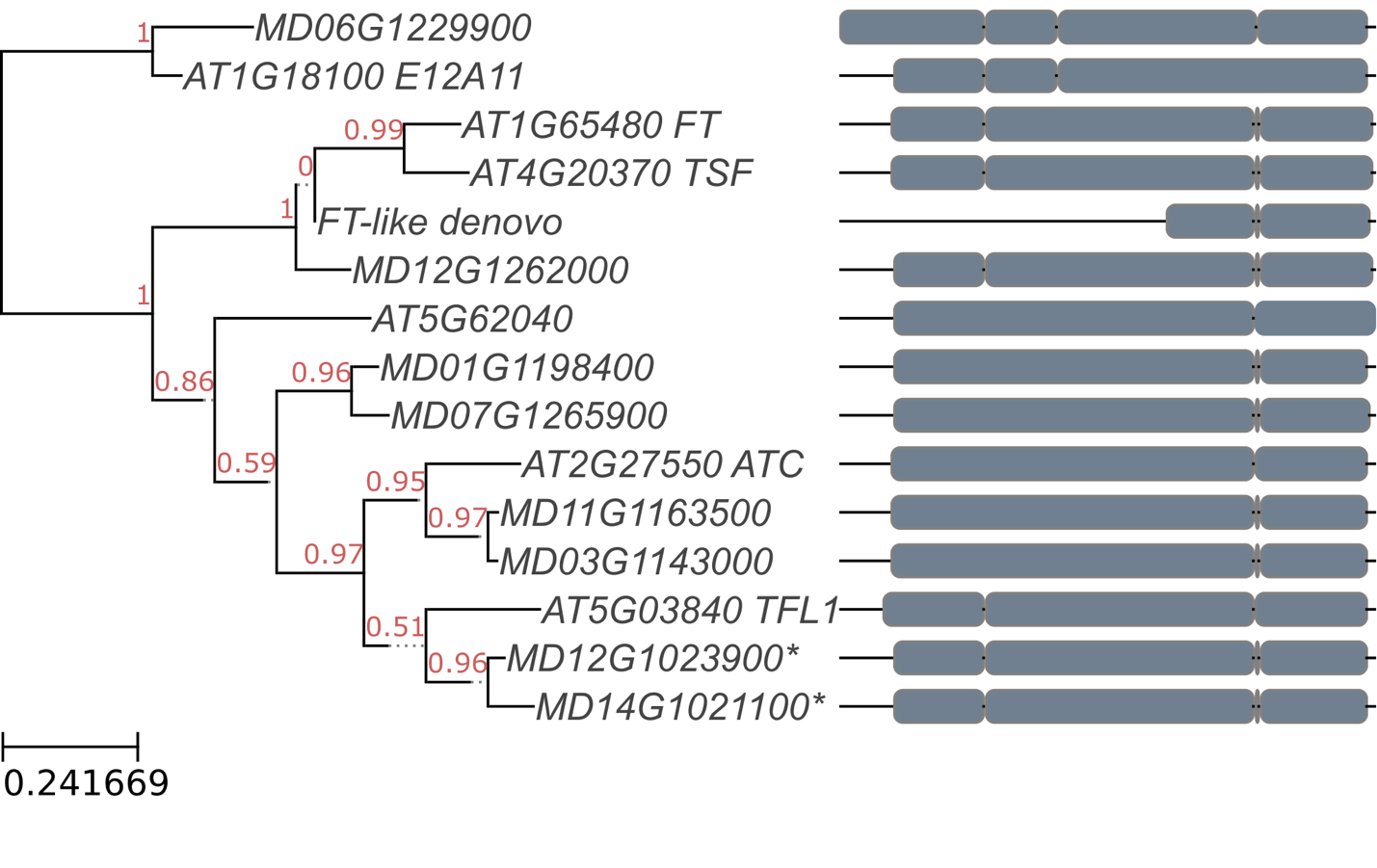


S2 Fig[n]. Phylogenetic analysis and sequence alignment for Arabidopsis and apple proteins most closely related to Arabidopsis *TFL1*. * Indicates reciprocal blast homolog.
