# Supplementary material for: Genetic mechanisms associated with floral initiation and the repressive effect of fruit on flowering in apple (Malus x domestica Borkh)
Source: PLoS One. 2021 Feb 19;16(2):e0245487. doi: 10.1371/journal.pone.0245487 (PMC7894833; doi:10.1371/journal.pone.0245487)
Supplement: S1 File — (DOCX) [file pone.0245487.s003.docx]

doi:10.5061/dryad.fn2z34tr5
